# The parable of arable land: Characterizing large scale land acquisitions through network analysis

**DOI:** 10.1371/journal.pone.0240051

**Published:** 2020-10-13

**Authors:** Roberto Interdonato, Jeremy Bourgoin, Quentin Grislain, Matteo Zignani, Sabrina Gaito, Markus Giger

**Affiliations:** 1 Cirad, TETIS, Montpellier, France; 2 TETIS, Univ. of Montpellier, APT, Cirad, CNRS, INRAE, Montpellier, France; 3 Dipartimento di Informatica, Università degli Studi di Milano, Milan, Italy; 4 Centre for Development and Environment (CDE), University of Bern, Bern, Switzerland; The Bucharest University of Economic Studies, ROMANIA

## Abstract

Land is a scarce resource and its depletion is related to a combination of demographic and economic factors. Hence, the changes in dietary habits and increase in world population that upturn the food demand, are intertwined with a context of increasing oil prices and rise of green capitalism that in turn impacts the demand in biofuel. A visible indicator of these phenomena is the increase, in recent years, of Large Scale Land Acquisitions (LSLAs) by private companies or states. Such land investments often lead to conflicts with local population and have raised issues regarding people’s rights, the role of different production models and land governance. The aim of this work is to show how publicly available data about LSLAs can be modeled into complex network structures, thus showing how the application of advanced network analysis techniques can be used to better understand land trade dynamics. We use data collected by the Land Matrix Initiative on LSLAs to model three land trade networks: a *multi-sector* network, a network centered on the *mining* sector and a network centered on the *agriculture* one. Then we provide an extended analysis of such networks which includes: (i) a structural analysis, (ii) the definition of a score, namely *LSLA-score*, which allows to rank the countries based on their investing/target role in the land trade network, (iii) an analysis of the land trade context which takes into account the *LSLA-score* ranking and the correlation between network features and several country development indicators, (iv) an analysis centered on the discover and analysis of network motifs (i.e., recurring patterns in the land trade network), which provides insights into complex and diverse relations between countries. Our analyses showed how the land trade market is massively characterized by a Global North-Global South dynamic, even if the investing power of emerging economies also has a major impact in creating relations between different sub-regions of the world. Moreover, the analyses on the mining and agriculture sectors highlighted how the role of several countries in the trade network may drastically change depending of the investment sector, showing diverse hierarchies between investor, intermediate and target countries.

## Introduction

Demand for land embodied in trade of mineral, fossil fuels and agricultural commodities has been shown to be very significant. It has been shown that, on balance, this trade results in displacement of “virtual water” and embodied land from poorer to richer countries [1–3]. The commodification of arable land and its entanglement within globalized trade networks have also largely been studied in the past few years [4, 5]. This provides the background on which the recent wave of large-scale land acquisitions (LSLA) evolved. The global financial and food crisis that took place in the years 2007/2009 gave rise to a series of events with a significant impact on global markets [6]. The political and economic instability created by the crisis generated a worldwide increase in food prices, and resulted in an increase in food insecurity and hunger in a large number of countries. In this context, it was witnessed a sudden increase in large-scale national and transnational commercial land transactions, or LSLA [7], which, though not being new as a phenomenon in absolute terms, received, in this moment, an increasing attention [8]. The phenomenon of LSLAs is highly controversial, and it is at the basis of many debates about people’s rights, production models, land governance, and may often lead to conflicts with local populations [9]. One of the main reasons for controversies is the debate between the proponents, highlighting how these large scale investments can leverage valuable economic opportunities [10], and a large body of authoritative opinions by authors and Non-Governmental Organizations (NGOs) suggesting that beside these opportunities may lie serious risks of corruption, and important threats to the rural poor’s livelihoods, e.g., loss of land and a progressive marginalization [11–13]. Recent research work decries that LSLAs rarely entail fair negotiations with farmers [14], and that unfair trade arrangements can be qualified as new manifestation of colonial power asymmetries between the Global North and the Global South [15]. While rural communities have historically lived with insecure land rights, nowadays the rate of large-scale acquisitions is increasingly jeopardizing people’s access to land [14, 16]. A rich number of research results has provided insights on the social, economic, and environmental impacts of large-scale international land acquisitions [17–28]. A meta-analysis of 44 scientific case studies conducted by [29] showed adverse livelihood impacts in many cases, which only in a limited number of cases was mitigated by positive impacts such as job creation. In this regard, a main issue found is that of the lack of transparency in land deals. In many cases, contracts between investors and host countries are not made public, and details such as the exact conditions of the contracts and the exact location of the contracted land remain unknown.

For this reason, providing consistent and reliable data on the different forms of deals, and on the dynamics of such phenomenon, is a challenging task that answers to an actual and urgent need [30]. Currently, in many countries land deals are under-regulated by public policies [21]. Well-established procedures may not exist, or lack implementation due to limited political will or practical means, or, also, procedures may be voluntary and non-binding (e.g., Free Prior and Informed Consent, Voluntary Guidelines on the Responsible Governance of Tenure). For a given country, official data sources and unofficial ones (e.g., assessments performed by NGOs) may often be inconsistent, and none of them may represent an accurate representation of the real situation [31]. Data however has been collected systematically by the Land Matrix Initiative since 2009. This database can considered to be the most comprehensive on LSLAs [32]. It is maintained by a consortium of research and development partners. The database is a structured collection of information coming from several different sources, such as government data, public press, scientific publications and voluntary contributions from specific individuals or institutes. In order to maintain a specific focus on *large scale* land acquisitions, only deals accounting for more than 200 hectares of leased or sold land are included in the database [16]. The database focuses on agricultural international investments, but increasingly covers domestic deals and other sectors such as mining and forestry.

Even though contributions to the Land Matrix database are numerous and heterogeneous, and that the number of listed land deals is constantly increasing, such a collection cannot still be considered exhaustive. The Land Matrix acknowledges that some data biases are unavoidable, given differences in government policies regarding data openness, freedom and strength of press, presence of active farmers organizations or existence of dedicated land observatories, or specific scientific studies, having been undertaken [22]. Land Matrix data has been used in numerous studies, focusing on different topics such as water [33, 34], the commons [14, 35], food security [36], labour markets [37], and biofuels [38]. For our topic of specific interest is the work on determinants of land acquisitions [39, 40]. Both Arezki et al. [39] and Lay and Nolte [40], using a gravity model approach, already found that LSLA are often related to weak governance systems and overlapping land rights, and that rich investor countries target poorer economies with abundant land and water resources. Authors in [41] however also showed that in many cases, investors aim at areas which are already supporting a considerable populations density, hence completion of land resources is likely to follow. Nevertheless, data science techniques have rarely been used to extract new knowledge from the database. In such a complex scenario, the application of data mining and network analysis techniques would allow to better characterize relations among countries and help to understand the dynamics of the land trade market. In literature, only a couple of works can be found that propose approaches in this direction. Seaquist et al. [42] combined data from the Land Matrix and GRAIN (www.grain.org, another LSLA database compiling information on deals from press articles and scientific publications) in order to build a land trade network, which is then analyzed focusing on its basic structural characteristics (e.g., betweenness, clustering coefficient, assortativity). However, the version of the Land Matrix database used in our work (database retrieved on 16th January 2020 on https://landmatrix.org) includes three times the number of entries with respect to the version analyzed in [42], which dates back to 2012, i.e., 2, 931 transnational deals vs 1, 006, making most analyses obsolete. Furthermore, data quality has increased significantly since its initial years [22], thanks to feedback from various side and new research results becoming available. Moreover, while the authors in [42] carried out their analysis by leveraging on basic network analysis measures, in this work we propose a wider study, which includes the introduction of a novel score (*LSLA-score*) specifically conceived for the task at hand, the application state-of-the-art techniques to discover network motifs corresponding to statistically significant land trade schemes, the comparison between network features and country development indicators, and the analysis of specific sub-markets. Mechiche-Alami et al. [43] recently proposed a study about transnational land acquisitions, focusing on the identification of different phases of activity in the land trade market during the years. The aim of their study is more to identify general trends that characterize different temporal phases of the land trade market, while in this work we want to focus on complex relations among countries, by also considering heterogeneity of the deals, namely the intention of investment and the implementation status. In [44], we published some preliminary results regarding the use of network analysis techniques for the analysis and characterization of LSLAs phenomena. The aim of this work is to push forward this promising approach based on complex network analysis, by providing a wider quantitative and qualitative analysis structured in the following main contributions:

- the modeling of three land trade networks out of the Land Matrix Initiative database: a *multi-sector* network, a network centered on the *mining* sector, and a network centered on the *agriculture* one;
- the definition of a score, namely *LSLA-score*, which allows to rank the countries based on their investing/target role in the land trade network;
- a structural analysis of the three land trade networks;
- an analysis of the land trade context which takes into account the *LSLA-score* ranking and the correlation between network features (i.e., both classic centrality measures and the *LSLA-score*) and several country development indicators;
- an analysis centered on the discover and analysis of network motifs (i.e., recurring patterns in the land trade network), which provides an insight into statistically significant land trade schemes, and by consequence into complex relations between countries;

The rest of the paper is structured as follows: Section 1 describes network modeling, discusses data and limitations aspects, and introduces an analysis of structural characteristics of the land trade networks, in Section 2 we introduce the *LSLA-score* and we use it to analyze the land trade context, in Section 3 we present our analysis based on network motifs, while Section 4 concludes the work.

## 1 The Land Matrix land trade network

In this section we will first provide details on the directed network model used in this work, then we will provide details about how the Land Matrix data was used to extract the three directed land trade networks (i.e., the *multi-sector* one, the *mining* one and the *agriculture* one) and we will discuss eventual limitations of the proposed modeling approach.

### 1.1 Network modeling

The land trade network can be defined as a standard directed graph *G* = (*V*, *E*, *w*), where *V* is a set of nodes representing countries, *E* is a set of edges, where the existence of an edge (*u*, *v*) indicates that a company from country *u* has at least a land trade deal involving country *v* as target country, and w:E⇒R is an edge weighting function. The weight of an edge *w*(*u*, *v*) is defined as the total surface of land (in hectares) acquired by country *u* (i.e., companies located in country *u*) in country *v*. In this work, we will focus only on transnational land trade deals, i.e., we exclude the deals for which the investing company is based in the target country. Note that some land trade deals may be operated by more than one company and, by consequence, by more than one investing country (i.e., resulting in multiple edges in the network). In order to provide a reliable representation of the current land trade situation, we model the land trade network upon deals that are labeled as *in operation* in the database. We recall that the Land Matrix database also includes abandoned deals and deals currently in their startup phase (i.e., land has been acquired but the production phase has not started yet), that have been disregarded in our analysis. We also excluded deals corresponding to terminated contracts, whose land may currently be part of new deals.

### 1.2 Data and limitations

All the transnational land trade networks used in this work (i.e., the multi-sector one and the two sector-specific land trade sub-networks) have been generated by using the public Land Matrix Initiative database (https://landmatrix.org/data/). Main characteristics of these datasets are summarized in Table 1. The Python code, as well as the original Land Matrix data and the land trade networks used in the context of this work are publicly available online at https://gitlab.irstea.fr/roberto.interdonato/lsla-networkanalysis. The snapshot of the database used for this work has been downloaded on January 16th, 2020. The only filter applied at downloading time is the one about the *Deal scope* being *transnational*, while the subset of deals having *Current implementation status* equal to *In operation (production)* was selected in a post-processing phase. As concerns the *mining* and *agriculture* sector-specific sub-networks, a filter on the *Intention of investment* attribute has been added. More specifically:

- the *mining* land trade subnetwork has been obtained by taking into account only deals having *Mining* as *Intention of investment*;
- the *agriculture* land trade subnetwork has been obtained by taking into account only deals having an *Intention of investment* in one of the following categories: *Biofuels*, *Fodder*, *Food crops*, *Agriculture unspecified*, *Livestock*, *Non-food agricultural commodities*.

**Table 1:** Characteristics of the Land Matrix Initiative datasets. The total involved land refers to the total size of deals currently in operation for each dataset.

*Dataset*	*multi-sector*	*agriculture*	*mines*
*#deals*	2930	2109	386
*#deals in operation*	1609	1185	161
*oldest negotiation date*	1893	1893	1994
*most recent negotiation date*	2019	2019	2018
*total involved land (ha)*	48,356,791	20,747,183	5,613,993

Trading relations are modeled upon two attributes: *Top parent companies* and *Location 1: Target country*. *Top parent companies* reports the investing company for a given deal, together with its country of origin. Since in this work we carry out a country-centered analysis (i.e., each node in our land trade network represents a country), we only take into account the country of origin, disregarding the information about the specific investment company (nevertheless, the use of such information may open interesting analysis scenarios, and is left as future work).

Please note that in the Land Matrix database more than one investing company may be listed under the *Top parent companies* attribute. In these cases, when these companies are based in different countries, we considered the deal as belonging equally to each country with the same (total) deal size. While this assumption introduces an overestimation in the edge weights, it is, in our opinion, the most reasonable one in our analysis scenario. Detailed information about the participation of each company in a given deal (i.e., percentage of investment) is not available. Here we chose to consider that a given parent company (and thus a given investing country) is involved in a deal of a certain total size with a given target country, regardless of the actual percentage of investment. This choice was rationalized by the following. Firstly, we used a public database without introducing third-party information that may have been difficult to retrieve and that would not have been available in all cases. Secondly, we oriented our analysis toward considering global trading relations. An alternative solution may have been to divide the total deal size over the number of top parent companies: while this may be reasonable, it would introduce a strong underestimation of the importance of certain deals when multiple companies are involved, which may lead to counter-intuitive results in our scenario. The same assumption has been made in presence of multiple values in the *Intention of investment* attribute, i.e., deals reporting multiple values have been considered has equally belonging to all the intentions of investment. The reason for this choice is the same as in the case of multiple investing countries, i.e., the choice to only use the public database without introducing third-party information that may be difficult to retrieve and not applicable to all cases.

As already mentioned, target countries are identified through the *Location 1: Target country* attribute of the database. While more than one location may be listed in the database for certain deals (the database includes attributes from Location 1 to Location 21), in all cases (as verified on the snapshot used for this work) they all belong to the same target country, i.e., the attributes from Location 2 to Location 21 (where not null) always refer to different areas in the same target country.

Please note that the Land Matrix Initiative database is constantly evolving, so that the analyses carried out in this work may give slightly different results with newer (more recent) versions of the database. Nevertheless, while the main focus of this work is in the modeling and characterization of global trading relations between countries, we are confident that the general outcomes of our analysis will stay significant even in presence of updates in the database.

### 1.3 Structural characteristics of the land trade networks

Before delving into the high-order structural properties of the land trade networks, we here discuss their main topological characteristics, summarized in Table 2, to get an overall picture of how the land deals among the countries are structured.

**Table 2:** Structural characteristics of the land trade networks.

*network*	*#nodes*	*#edges*	*reciprocity*	*avg_path_length*	*avg_cc*	*transitivity*	*assortativity*
*multi-sector*	142	686	0.01	2.59	0.13	0.06	−0.09
*agriculture*	135	522	0.003	∞(2.77)	0.11	0.04	−0.07
*mining*	66	142	0.0	3.09	0.05	0.01	−0.14

#### 1.3.1 The multi-sector land trade network

First, we inspected some characteristics related to the connectedness and the local clustering of the network. Specifically, we computed the average path length on the undirected counterpart of the trade network, i.e. the average length of the minimum shortest among each pair of nodes in the network. A relatively low average path length on the undirected graph (2.59) indicates that the land trade network is rather compact, i.e. on average a pair of nodes is separated by two other country nodes. Furthermore, to evaluate the local propensity of forming clustered structures around nodes, we computed the average clustering coefficient, i.e. the average over the local clustering coefficient of each node. The local clustering coefficient of a node is the ratio between the actual links between its neighbors and the number of possible pairs between them. An average clustering coefficient (computed on the undirected network) of 0.13 indicates a certain propensity of creating transitive connections among the neighbors of node.

Second, we evaluated the level of asymmetry of the links by measuring the link reciprocity, i.e. probability that if we observe the directed link (*i*, *j*) we will also observe the reciprocal link (*j*, *i*). The network shows an extremely low reciprocity (0.01), showing how it is quite uncommon to have reciprocal investments in land deals between countries. This low value is not surprising, and can be seen as a quantitative assessment of an asymmetry in the land trade network which can be considered as a direct heritage of the colonial power. The asymmetry of the land trade market also impacts on the number of strongly connected node pairs, i.e. node pairs (*i*, *j*) which are connected by a directed path from *i* to *j* and vice versa. The low percentage of strongly connected node pairs (16%, for a directed average path length of 2.91), confirming that bidirectional paths are uncommon in our scenario.

Summing up, the multi-sector land trade network is characterized by a strong asymmetry of the deals which accounts for an high heterogeneity of the buying capacity of the countries. This asymmetry impacts the chains (paths) of deals which are *i)* quite short and *ii)* introduce a sort of rank among the countries involved in the chains. Furthermore, circular chains of deals are not rare and deserve further investigation. In fact, in Section 3, we will investigate on high-order structures by performing an extensive analysis of network motifs of size 3, 4 and 5, with the aim to deepen our knowledge about land trade schemes among countries.

#### 1.3.2 The *agriculture* and *mining* land trade networks

Looking at the structural characteristics of the *agriculture* and *mining* land trade networks reported in Table 2, it can be noted how both networks show substantial differences with respect to the multi-sector one. A first outcome (relatively expected, due the lower number of considered deals, and, by consequence, of links) is that both subnetworks are less dense than the multi-sector one. Both networks show a higher average path length than the multi-sector one, i.e., 3.09 for *mining*, and 2.77 for the largest connected component of *agriculture* (the *agriculture* network in fact is not connected, resulting in an infinite average path length, due to a disconnected component represented by the Barbados and Guyana countries). Such lower density is confirmed by lower values of clustering coefficient and transitivity with respect to the multi-sector network, with the *mining* network appearing less dense than the *agriculture* one.

It is interesting to note how the percentage of reciprocal edges, already low in the multi-sector case, is even lower in these networks, and even zero in the *mining* case. This confirms once more the separation between investing and target countries, and emphasizes how such target countries are indeed richer in terms of exploitable resources. Such stronger division between investing and target countries (with respect to the multi-sector case) is also supported by the fact that both subnetworks are more disassortative than the multi-sector one, i.e., nodes in these networks have a lower tendency to connect with nodes with similar structural characteristics, which translates into a lower tendency to be involved in deals with countries with similar profiles. In particular, the *mining* network is slightly disassortative—with an assortativity coefficient of −0.14—which highlights the divide between North and South (and more in general between *poor* and *rich* countries). The differences in the *agriculture* and *mining* land trade mechanisms (between them and with respect to the multi-sector case) will be confirmed, and analyzed in detail, thanks to quantitative and qualitative analyses based on network motifs (cf. Section 3).

## 2 Analysis of the land trade context

### 2.1 The *LSLA-score*

The modeling of the Land Matrix database as a directed weighted network allows us to provide a rank of the countries based on their status of investing/target countries in the context of large scale land acquisition, we here provide a score, namely the *LSLA-score*, based on the total surface of acquired and sold land for each country, as reported in the Land Matrix database. Note that in this work we will refer to *sold* land with a wide meaning, which includes contracts of different nature (e.g., leasing, rent, etc.).

The *LSLA-score* *s* for each country *u* in the land trade network is defined as follows:

$$\begin{array}{c} s\left(u\right)=\log_{10}(\frac{1+\sum_{(v,u)\in E}w\left(v,u\right)}{1+\sum_{(u,v)\in E}w\left(u,v\right)}) \end{array}$$ (1)

To prevent zero or infinite ratios, the values of *s*(⋅) are Laplace add-one smoothed. In other words, the *LSLA-score* is proportional to the weighted in/out-degree ratio of each country, i.e., the ratio between acquired and sold land for each country. By consequence, low values of *LSLA-score* will correspond to *investing* countries, while higher ones to *target* countries. Given the fact that for certain countries this ratio can be strongly unbalanced towards *investing* or *target* profiles, a logarithmic scaling is added in order to make the score distribution smoother. For ease of interpretation, in the following, all the *LSLA-score* values are rescaled using min-max normalization, in order to take into account values of *s*(⋅) in the range [0, 1].

### 2.2 The *multi-sector* land trade network

**Fig 1:**
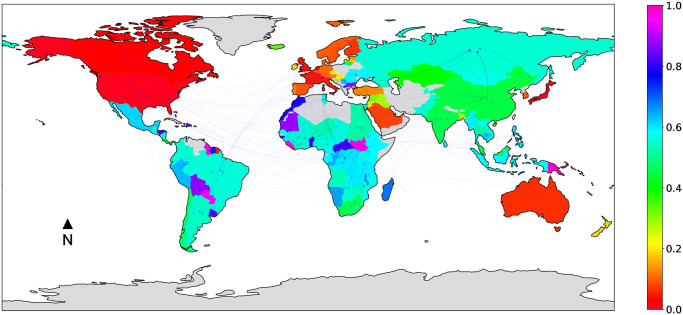
*Multi-sector* land trade network, where countries are colored based on their normalized *LSLA-score*, and edges opacity is proportional to edge weight.

Fig 1 provides a visualization of the multi-sector land trade network, where countries are colored based on their normalized *LSLA-score*, and edges opacity is proportional to edge weight (the maps were obtained using the *cartopy* and *networkx* python libraries). Recall that low values of *LSLA-score* (*red* countries in the map) correspond to big investors, while high values correspond to target countries (*blue* and *fuchsia* countries in the map).

As pictured by Fig 1, investments are globalised. Hence, a worldwide network of companies are investing in foreign lands. Mapping *LSLA-score* values allows us to easily identify investments profiles. A first acknowledgement is the obvious divide existing between North and South. Indeed, while it is easy to observe that investing countries can be found all over the world, an important correlation emerges between the size of deals in operation and the location of investors, which is highlighted by the values of *LSLA-score*. As a matter of fact, countries with a normalized *LSLA-score* close to 0.0 are mainly composed by the wealthiest and more powerful countries, for most of them, members of G20 and/or are strongly linked to Paris Club (see Table 3 for detailed memberships), a group of 22 creditor countries coordinating loans to countries in financial difficulties.

**Table 3:** Memberships of world countries to the G20 and Paris Club groups.

Country	G20	Paris Club	Country	G20	Paris Club
Countries	G20	Paris Club	Countries	G20	Paris Club
Australia		✔	Japan	✔	✔
Austria		✔	Mexico	✔	
Argentina	✔		Netherlands		✔
Belgium		✔	Norway		✔
Brazil	✔	✔	Russia		✔
Canada	✔	✔	Saudi Arabia	✔	
China	✔		South Africa	✔	
Denmark		✔	South Korea	✔	✔
Finland		✔	Spain		✔
France	✔	✔	Sweden		✔
Germany	✔	✔	Switzerland		✔
India	✔		Turkey	✔	
Indonesia	✔		United Kingdom	✔	✔
Ireland		✔	United States	✔	✔
Israel		✔	European Union	✔	
Italy	✔	✔			

In terms of target countries, comparing the different sides of Table 4 confirms the difficulty in obtaining a clear image of their concrete materialization, already reported at national level [45]. Between what is reported as contractually acquired land and what is referenced as in operation, we get a sense of the scale of investments within a country, knowing that precise and dynamic figures will never be accessible, especially at this global scale. In terms of magnitudes, this table allows acknowledging that target countries (normalized *LSLA-score* close to 1.0) remain Southern countries where appropriate climate conditions, profitable labor force, and land fertility allow countries to invest in production at competitive costs, with available arable land and water, and in a general context of weak agricultural infrastructure and margins for improving yields. This vision is supported by many development partners and hosting countries, often to the detriment of local practices portrayed as obsolete. Focusing on Liberia, which is the country showing the highest *LSLA-score*, a drastic increase in land deals dates back to period 2007-2008, with investments in agriculture and forestry from Asia (China, Malaysia, Singapore), Europe (UK, Luxembourg, Italy), and other West African countries (Ivory Coast, Nigeria), plus a major interest of Canadian investors in the mining sector. A main reason for this increasing rate of large-scale land concessions to private investors lies in the fact that, since the end of the civil war in 2003, Liberia has been struggling to enact a land law, which, coupled with weak land and natural resources management, contributed to tenure insecurity. A land reform process, which resulted in the establishment of a Land Commission, has been started by the Liberian government in 2009 with the aim to address this issue. Since then, the investment activity in the country seems to have drastically slowed down (according to the data registered by the Land Matrix).

**Table 4:** Top-10 countries for percentage of agricultural land acquired by foreign countries in transnational land trade deals considering all deals under contract (left table) and only deals currently in operation (right table).

*rank*	*Country*	*% land*	*rank*	*Country*	*% land*
1	Papua New Guinea	94%	1	Guyana	36%
2	Guyana	57%	2	Papua New Guinea	14%
3	Gabon	48%	3	Solomon Islands	7%
4	Liberia	47%	4	Uruguay	6%
5	Lao PDR	27%	5	Lao PDR	6%
6	Congo, Rep.	15%	6	São Tomé and Principe	5%
7	Cameroon	13%	7	Jamaica	4%
8	Sierra Leone	12%	8	Guatemala	3%
9	Congo, Dem. Rep.	11%	9	Ukraine	3%
10	São Tomé and Principe	10%	10	Liberia	2%

**Fig 2:**
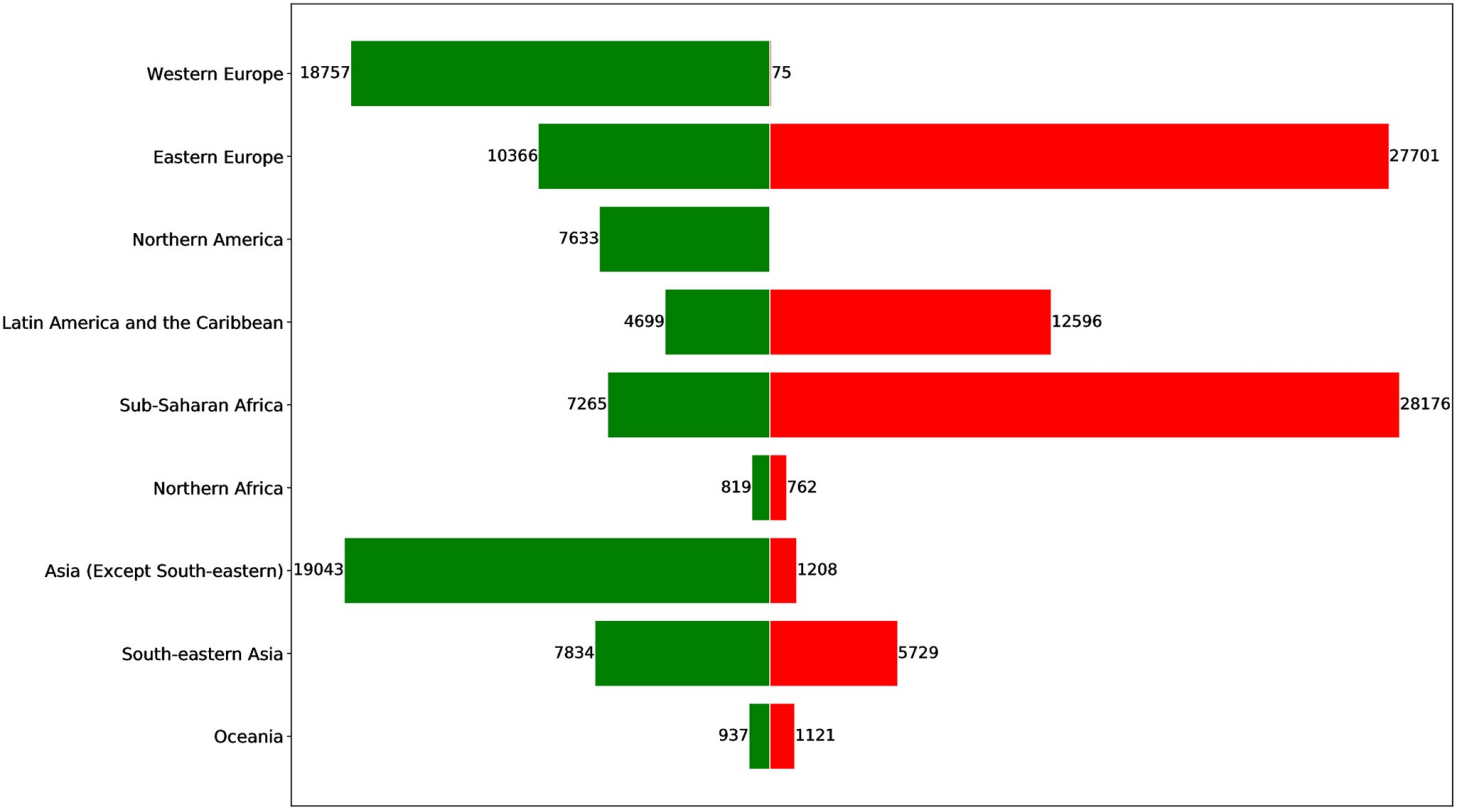
Barchart showing the quantity (thousands of hectares) of acquired land (weighted outdegree, green bar on the left) and sold/leased land (weighted indegree, red bar on the right), for different world sub-regions.

Fig 2 reports on the quantity (thousands of hectares) of acquired land (weighted outdegree, green bar on the left) and sold/leased land (weighted indegree, red bar on the right), for different world sub-regions. The sub-regions in the barchart are based on the geographic regions classification by the United Nations Statistics Division (UNSD) (https://unstats.un.org/unsd/methodology/m49/). More specifically, for ease of interpretation in the context of LSLA investment dynamics, all Asian sub-regions except *South-eastern Asia* were grouped in the *Asia (Except South-Eastern)* class, as well as all European regions except *Eastern Europe* were grouped in the *Western Europe* class. All other classes correspond either to continents (*Oceania*) or to sub-regions (the remaining classes) from the original UNSD classification. It can be noted how the values of *LSLA-score* showed in Fig 1 are consistent with the sub-region profiles on the global land market. Regions belonging to the Global North like *Western Europe*, *Northern America* and, to a lesser extent, *Asia (Except South-Eastern)*, confirm to be mainly composed by investing countries, that correspond to *source* nodes in the network (i.e., no incoming links, normalized *LSLA-score* around 0.0). Such leading countries in terms of global foreign direct investment, are highly ranked in terms of land investments [46]. In other regions a certain balance between the weighted indegree and outdegree can be noted, mainly due to the presence of the BRICS countries (Brazil, Russia, India, China and South Africa). As a results, regions belonging to the Global South like *Latin America and the Caribbean* and *Sub-Saharan Africa*, mainly composed by target countries (and thus corresponding to high quantities of leased/sold land), also show a significant total quantity of acquired land thanks to emerging economies like Brazil and South Africa. A similar observation can be drawn for *Eastern Europe*, dominated by the investing dynamics of the Russian federation.

**Fig 3:**
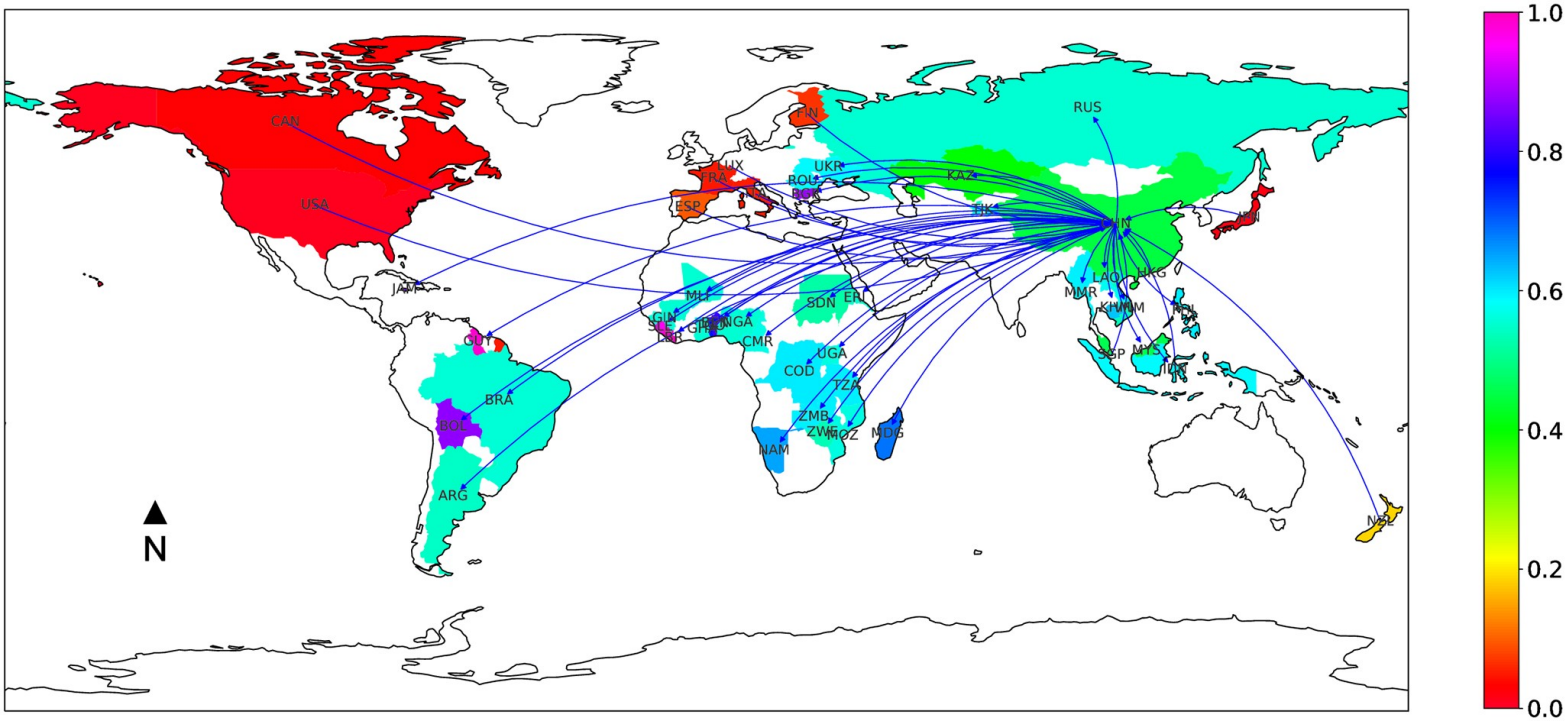
Focus on Chinese land trade network.

These emerging countries have important investments capacities, scarce arable lands and water, as well as increasing populations (e.g. India, China). As a result, China (through companies and State) is addressing its resource needs through investments in developing countries (Fig 3). Hence, China has been investing massively since 2010, mainly outsourcing agricultural production to Russia, Guyana, Congo Democratic Republic and Mozambique, targeting biofuel investments in Mali and Madagascar and investing in forestry in South-East Asia (Vietnam, Myanmar, Lao PDR, Cambodia and Indonesia).

**Fig 4:**
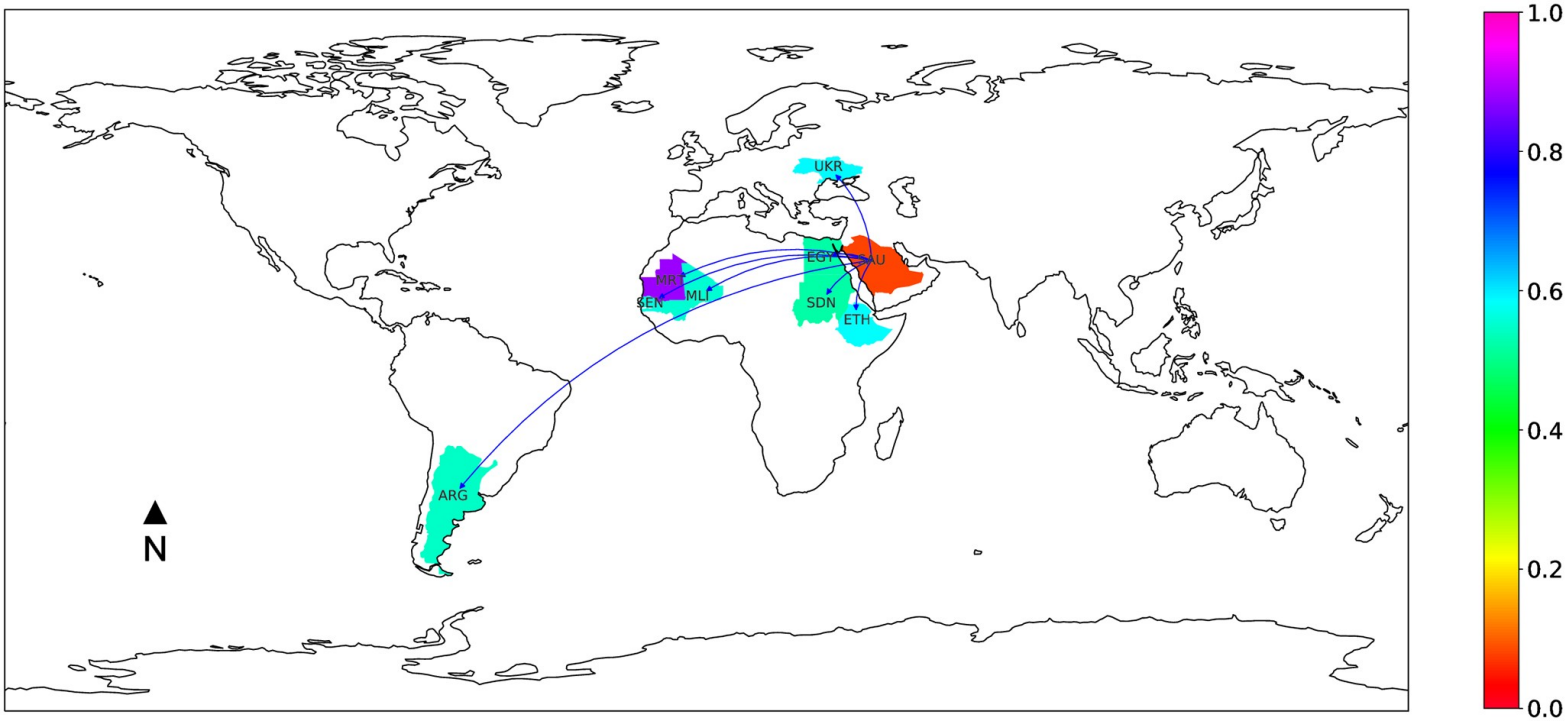
Focus on Saudi Arabia land trade network.

In addition to countries looking for resources, other countries are using trade to enforce other objectives. For instance, Saudi Arabia and the United Arab Emirates are facing structural deficits in agricultural land and water resources, and are highly dependent for food. While targeting resources of other countries, this countries have the dual aim of forging ties with Muslim African countries. This religious diplomacy is linked to the desire to spread a certain version of Islam throughout the world (see Principles of Da’awa, Basic Law 1992, art. 13). This international proselytism promoting a Salafist Islam is evolving in parallel of land investments [47]. The map in Fig 4 clearly shows that Saudi Arabia has its place in West Africa, and this influence will be consolidated by the financial support it has recently promised to the G5 Sahel force.

### 2.3 Land trade in the agricultural and mining sectors

In order to deepen our knowledge of the land trade context, in this section we carry out an analysis focused on two specific investment sectors: agriculture and mining. Development through growth, promoted since the end of the Second World War in 1945 and the enactment of the Marshall Plan, have been disparaged for a while [48], but has recently reached the masses, which ask for change [49]. Our current way of life, mainly based on petrol use is widely contested [50]. Globally, a wide consensus has emerged with a new watchword, the sustainability transition, which advocates for more sustainable modes of production and consumption, and has received increasing attention [51]. Discourses from industry leaders and politicians are embracing these rightful intentions, but are being used to restructure capitalism and promote new consumption patterns, instead of envisaging deep-structural changes in the system [52]. Indeed, proposed pathways for a so-called ecological transition are directed towards an energy transition that will see a major increase in mining rare earth element, which in return will have high negative environmental externalities [53]. We can then envisaged an increase in the mining sector in the coming years. Extractive industries are already targeted as a concrete threat to indigenous communities and many cases of land grabbing from indigenous peoples are already being thoroughly documented [54].

**Fig 5:**
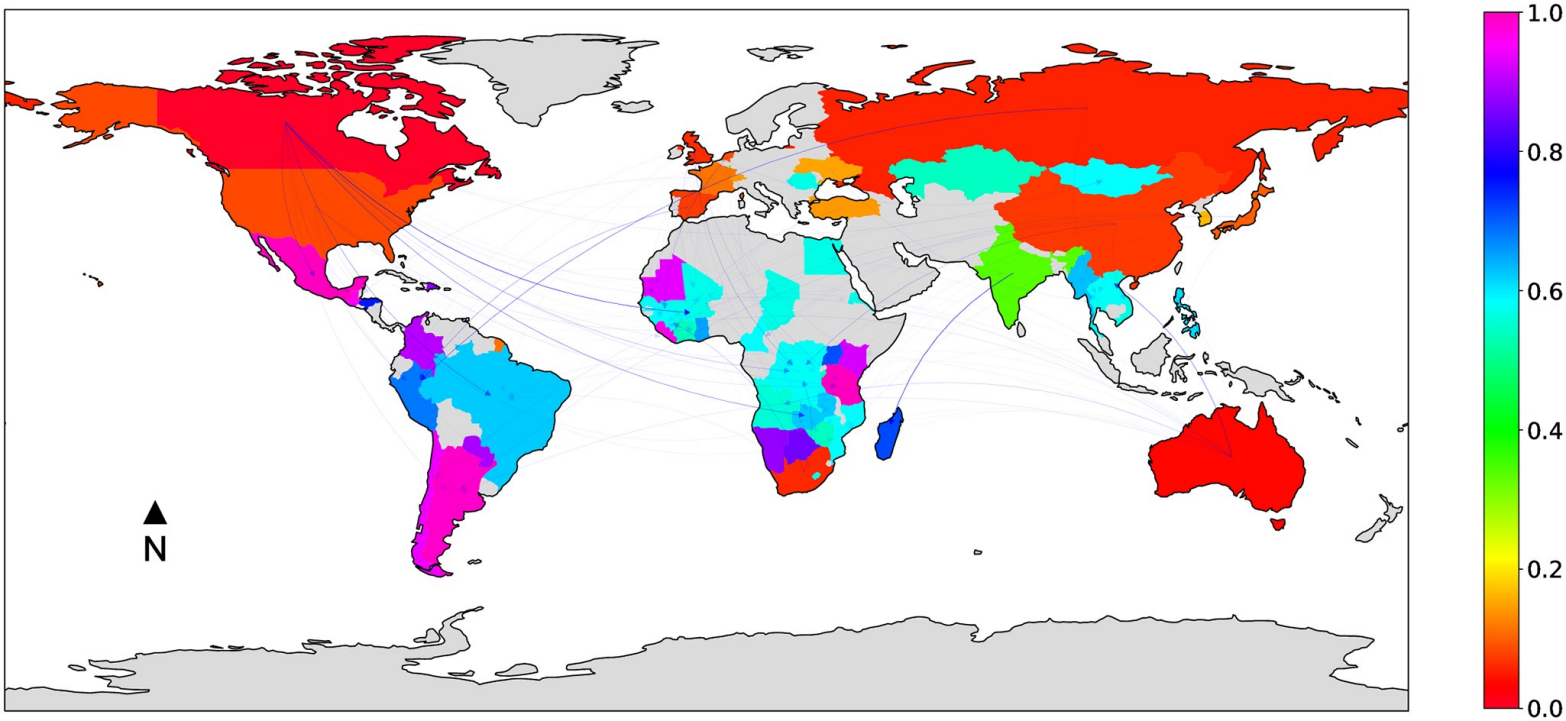
*Mining* land trade network, where countries are colored based on their normalized *LSLA-score*, and edges opacity is proportional to edge weight.

For these reasons, in the context of this work, we find it interesting to focus on the *mining* land trade network, which is reported in Fig 5 (with the countries colored according to their *LSLA-score*). The map shows that land deals are mainly located in Central America, South America, Africa and South Asia, where the Global North and BRICS are relocating extraction of minerals and related pollution. In comparison with the multi-sector land network (cf. Fig 1), we see very clear patterns and country profiles. For instance, Mexico, Argentina, Mauritania and Tanzania have a *LSLA-score* close to 1.0, which illustrates their propensity to attract investments. On the other hand, countries that in the multi-sector network have been viewed both as investors and as countries receiving investments, are designated only as investors in the mining sector. Here, we can highlight countries like Russia, South Africa, and to a lesser extent China (i.e., *LSLA-score* close to 0.0). Many countries in Africa or Middle East are not referenced as part of the mining network. Nevertheless, it is important to note that most developing countries are partly entangled in the network and attract investment, as much as they invest in other countries. With the race to rare earth elements that is accompanying the development of “green” energy, this baseline will help to monitor the dynamics of mining activities.

**Fig 6:**
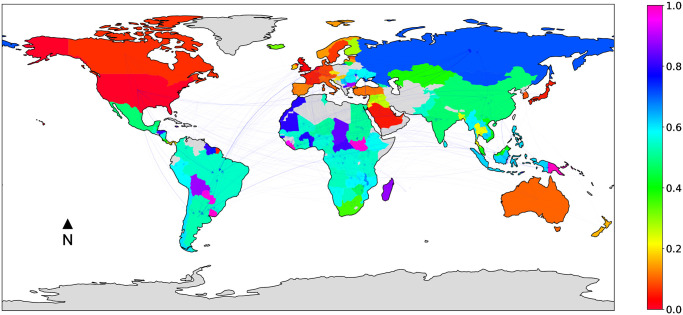
*Agriculture* land trade network, where countries are colored based on their normalized *LSLA-score*, and edges opacity is proportional to edge weight.

The second sector of investment we focus on is the agricultural one (Fig 6), which is also at the heart of the land trade network. Widespread narratives have identified the financial crisis of 2008 as the trigger for growing demand for agricultural lands, and concerns about scarcity as the spark for the global land rush [55]. Some authors still assert that foreign investment in agriculture are welcomed as an opportunity “to overcome decades of under-investments in the sector, create employment, and provide access to financial services and technology” [39] (pages 207–208). Ironically, this point of view is promoted by institutions that in previous decades imposed structural adjustment plans that contributed to the dismantlement of agricultural sectors in developing countries [56]. Promoting liberalism and land trade in the agricultural sector is now coming to fruition. Looking at the map in Fig 6 it can be noted how, unlike what was previously observed for the mining sector, the BRICS do not guide the investors and the network is led by countries from North America, Western Europe, some Gulf states (i.e. Saudi Arabia and the United Arab Emirates). For the latter, it highlights their structural deficit in agricultural lands and the current drastic state of water scarcity. For the former, high GNI countries, often former colonial powers, are perpetuating their presence in the Global South by appropriating resources. Regarding target countries, the network is displaying similar trends to the multi-sector network in Fig 1. Yet, we note that certain countries change patterns, being more involved in land acquisition (e.g., Thailand and Mexico), or becoming targets (e.g., Uruguay, Madagascar and Burkina Faso).

### 2.4 Correlation with country development indicators

In order to take a further step towards the characterization of land trade profiles, we here provide an analysis on the multi-sector land trade network which aims at studying the correlation between network features (i.e., standard centrality measures) and several country development indicators, which encompass the social, environmental, economic and governance dimension in each country. The indicators taken into account in our analysis are the following:

- **Biocapacity**. It is an indicator provided by the Global Footprint Network [57]. It consists in the area of productive land available to produce resources or absorb carbon dioxide waste, given current management practices. It is measured in standard units called global hectares.
- **Worldwide Governance Indicator (WGI)**. This indicator reports aggregate and individual governance indicators for over 200 countries and territories over the period 1996–, for six dimensions of governance: voice and accountability, political stability and absence of violence, government effectiveness, regulatory quality, rule of law and control of corruption [58].
- **Corruption Perceptions Index (CPI)**. It is an indicator provided by Transparency International [59]. It consists in a rank of 180 countries and territories by their perceived levels of public sector corruption according to experts and business people. A scale from 0 to 100 is used, where 0 corresponds to highly corruption and 100 to very clean countries. More than two-thirds of countries score below 50 on this year’s CPI, with an average score of just 43.
- **Human Development Index (HDI)**. This indicator was created to emphasize the role of people and their capabilities as the ultimate criteria for assessing the development of a country, in opposition to classic indicator which tend to focus solely on the economic growth. It is a statistic composite index of life expectancy, education, and per capita income indicators [60].
- **Growth National Income (GNI)**. Calculation of the GNI is based on the total income earned by a nation’s people and businesses, including investment income, regardless of where it was earned [61]. It also covers money received from abroad such as foreign investment and economic development aid.
- **Global Food Security Index (GFSI)**. This indicator defines vulnerability to food insecurity by aggregating drivers of food security across 113 countries [62]. Since 2012, food security specialists have been consulted by The Economist Intelligence Unit to assess indicators related to affordability, availability, quality & safety, and natural resources & resilience. Data for the quantitative indicators are drawn from national and international statistical sources.

**Fig 7:**
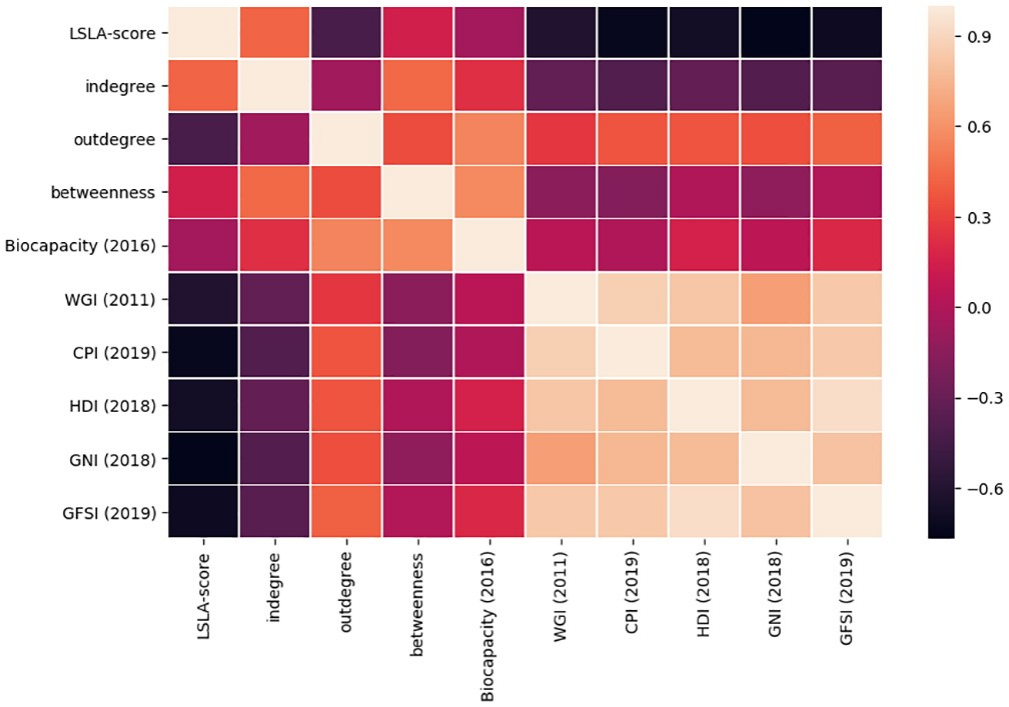
Heatmap showing the correlation (Pearson correlation coefficient) of *LSLA-score*, indegree, outdegree and betweenness centrality of the land trade network with different country development indicators.

Fig 7 shows a heatmap reporting the correlation (Pearson’s correlation coefficient) of the *LSLA-score*, *indegree*, *outdegree* and *betweenness* centrality of the land trade network with respect to such indicators. The heatmap was produced using the *seaborn* python library. It is easy to observe how land deals are directed towards countries experiencing low development rates (economic, social, governance). This is highlighted by the negative correlation of both *LSLA-score* and *indegree* with the last five indicators (WGI, CPI, HDI, GNI, GFSI). This scenario is more evident in the case of *LSLA-score*, showing correlations in the range −0.77 ≤ *ρ* ≤ −0.61, while *indegree* shows correlations in the range −0.39 ≤ *ρ* ≤ −0.31. This confirms once more how the *LSLA-score* is a valid indicator in the context of large scale land acquisitions, proving to be more informative than the indegree alone (i.e., corresponding to the total quantity of acquired land). Conversely, investing countries are characterized by higher CPI, HDI and GNI, with positive correlations between *outdegree* and such indicators in the range 0.34 ≤ *ρ* ≤ 0.37.

Focusing on *biocapacity*, it can be noted how investing countries (i.e., low values of *LSLA-score*) tend to show higher quantities of productive land (i.e., higher levels of *biocapacity*) than target ones. We can infer that the *biocapacity* indicator somehow serves as a proxy for measuring the direct negative impact on the environment produced by the common practice to outsource primary production to foreign countries. Another result that is worth discussing is the high positive correlation (*ρ* = 0.57) between *biocapacity* and *betweenness*, i.e., a centrality measure based on the number of shortest paths passing through a certain node. A possible interpretation of this correlation is that the countries characterized by high values of biocapacity play the role of *flow hubs* in the land trade network. Following this observation, we can infer that: (i) even though these countries have a significant quantity of their land leased or sold in transnational deals, their economic power is enough to allow them to invest in turn in the acquisition of foreign land, (ii) the land trade deals involving such flow hubs are likely to belong to distinct markets, i.e., countries involved in their ingoing and outgoing investments are not likely to be connected between them.

**Fig 8:**
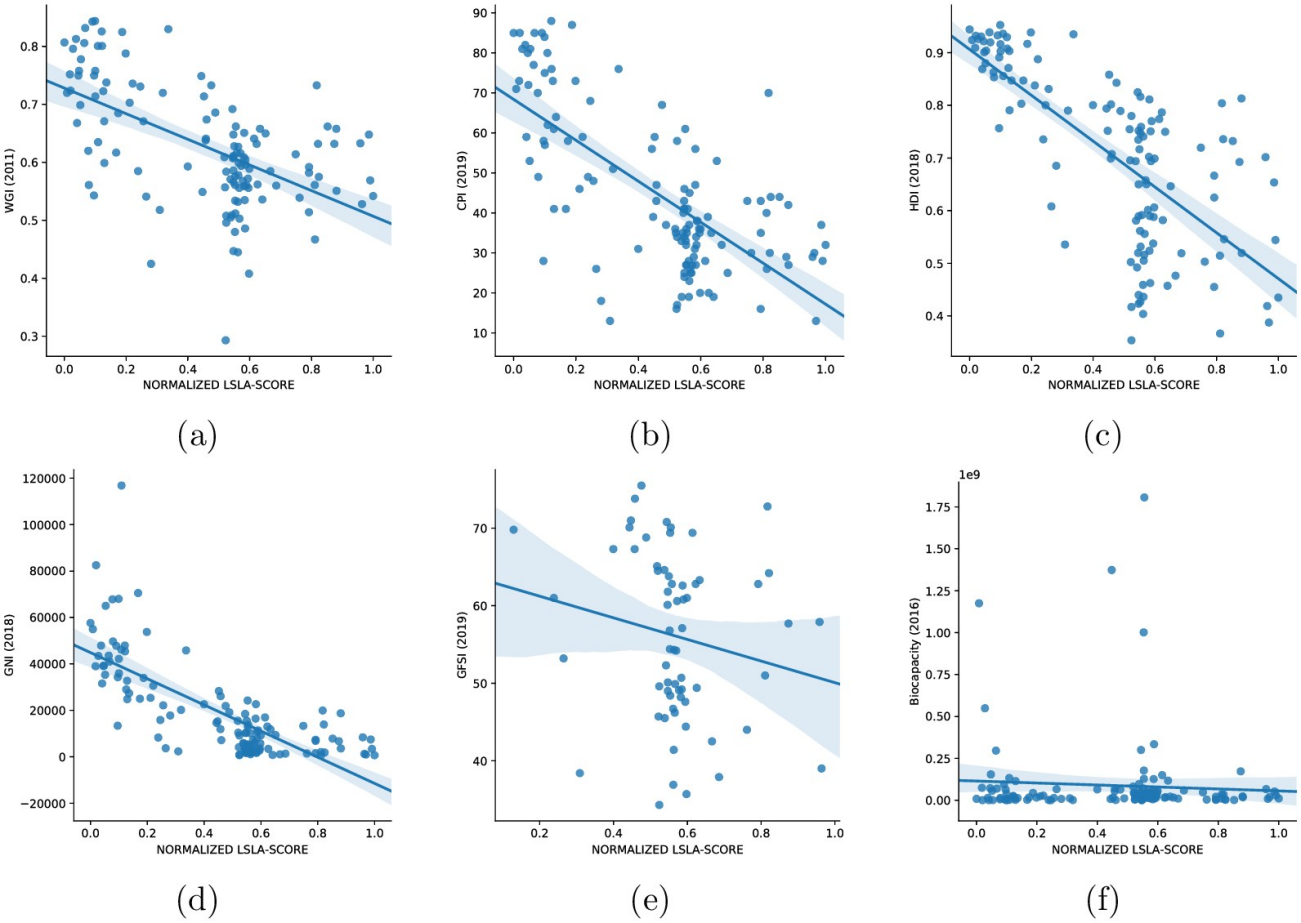
Scatter plots showing the distribution of *LSLA-score* with respect to the six country development indicators: WGI (a), CPI (b), HDI (c), GNI (d), GFSI (e) and Biocapacity (f).

Cross-referencing target countries and food insecurity provides interesting insights. The *global food security* indicator (GFSI) shows great correlation between investments high-value targets and countries most affected by malnutrition or famine. One could think investments are a direct response to such contexts, and that attracting investments would be designed by national governments to contribute to alleviate food insecurity and foster rural development though trickle-down economics [63]. Unfortunately, addressing national food security issues is rarely the mandate of transnational companies and food insecurity is seldom alleviated by production because companies are more likely to export or grow non-food crops (e.g. biofuel) [64]. Mining is also booming with the growing demand for mining goods in the current context of energy transition [53]. It is causing loss for land that could have been devoted to food crops, and increase in agricultural products’ prices on local markets. Access to food is also more difficult for local populations due to the loss of agricultural sovereignty. This situation often leads to tensions between elite owners and small producers and eventually to political instability. Cross analyzing the indices provides a clear message that is backed by meta-analyses released by the World bank [65]. Investors choose states where the application of norms is weak, where institutions are most “corrupt or indebted governments with little ability to regulate the transaction or prevent buyers from targeting the poorest rural communities, expelling people with non-traditional land title from their land” [7] (page 210). Fig 8 highlights this situation by showing the distribution of *LSLA-score* with respect to the six development indicators took into account. It is easy to see how in most cases the scatter plots illustrate this narrative and tendency with a clear significance towards the fact that investments are targeting the poorest countries experiencing the weakest levels of governance and the highest levels of corruption. This tendency can easily be noted for WGI, CPI, HDI and GNI (Fig 8(a)–8(d)) and, to a lesser extent, in GFSI (Fig 8(e)), which shows a narrower distribution centered around medium *LSLA-score* values. Regarding biocapacity (Fig 8(f)), this indicator seems to assume low values for most countries, but several outliers corresponding to countries with high biocapacity and medium/low *LSLA-score* can be easily identified, supporting the previous observations about this indicator.

## 3 Analysis based on network motifs

We move from a node-centric approach in the analysis of the land trade networks to an approach based on high-order structures. Specifically, we focus on network motifs, i.e. induced subgraphs that occur significantly more often in an observed network than would in a randomized one with identical properties. Network motifs represent a powerful tool to discover the existence of underlying non-random structural or evolutionary design principles that might have affected the growing of the network. The idea of network motifs was introduced by Milo *et al*. [66] in biological networks, with the aim to identify recurrent over-represented patterns of interaction, which may correspond to the functional or organizational building blocks of the network. Recently, such a tool proved to be effective in the analysis of different kinds of networks, since the concept of characterize a network by studying its basic building blocks is relatively easy to generalize. For instance, network motifs, even temporal annotated, have been used to characterize homophily and information cascades in social networks, communication patterns, and also to perform fraud detection in financial networks or to identify special connections among firms in economic and financial networks. The motivation that encouraged us to perform an analysis of the land trade network based on network motifs is that this will allow to discover higher order correlations between the entities (i.e., investing and target countries), thus providing new insights about the dynamics of large scale land acquisition phenomena.

In details, we analyze directed 3–, 4– and 5–network motifs on the directed land trade networks, neglecting the edge weights. Since the networks are relatively small in size, we apply an exact network motif algorithm in order to calculate the census of all subgraphs of size 3, 4 and 5. More specifically, we adopt the state-of-art solution proposed in [67] based on *g-tries* to do the census of all the *k*-subgraphs in the original network and in the random ones used to assess the significance of each subgraph. We generate 100 random networks preserving the in and out degree and we measure the statistical significance of a subgraph *D*_*k*_ by its *z-score* *Z*:

$$\begin{array}{c} Z\left(D_{k}\right)=\frac{f{\left(D_{k}\right)}^{G}-{\hat{f}}_{rand}\left(D_{k}\right)}{\sigma (f_{rand}\left(D_{k}\right))} \end{array}$$ (2)

where *f*(*D*_*k*_) represents how many times the subgraph *D*_*k*_ occurs in the network *G*, while ${\hat{f}}_{rand}$ and *σ*(*f*_*rand*_) correspond to the mean and standard deviation of the frequency of *D*_*k*_ in the random networks, respectively. Since we are interesting in over- and under-represented network motifs, we focus our analysis on network motifs with |*Z*(*D*_*k*_)| ≥ 1 and *f*(*D*_*k*_)^*G*^ ≥ 4.

### 3.1 Motifs on the multi-sector network

**Fig 9:**
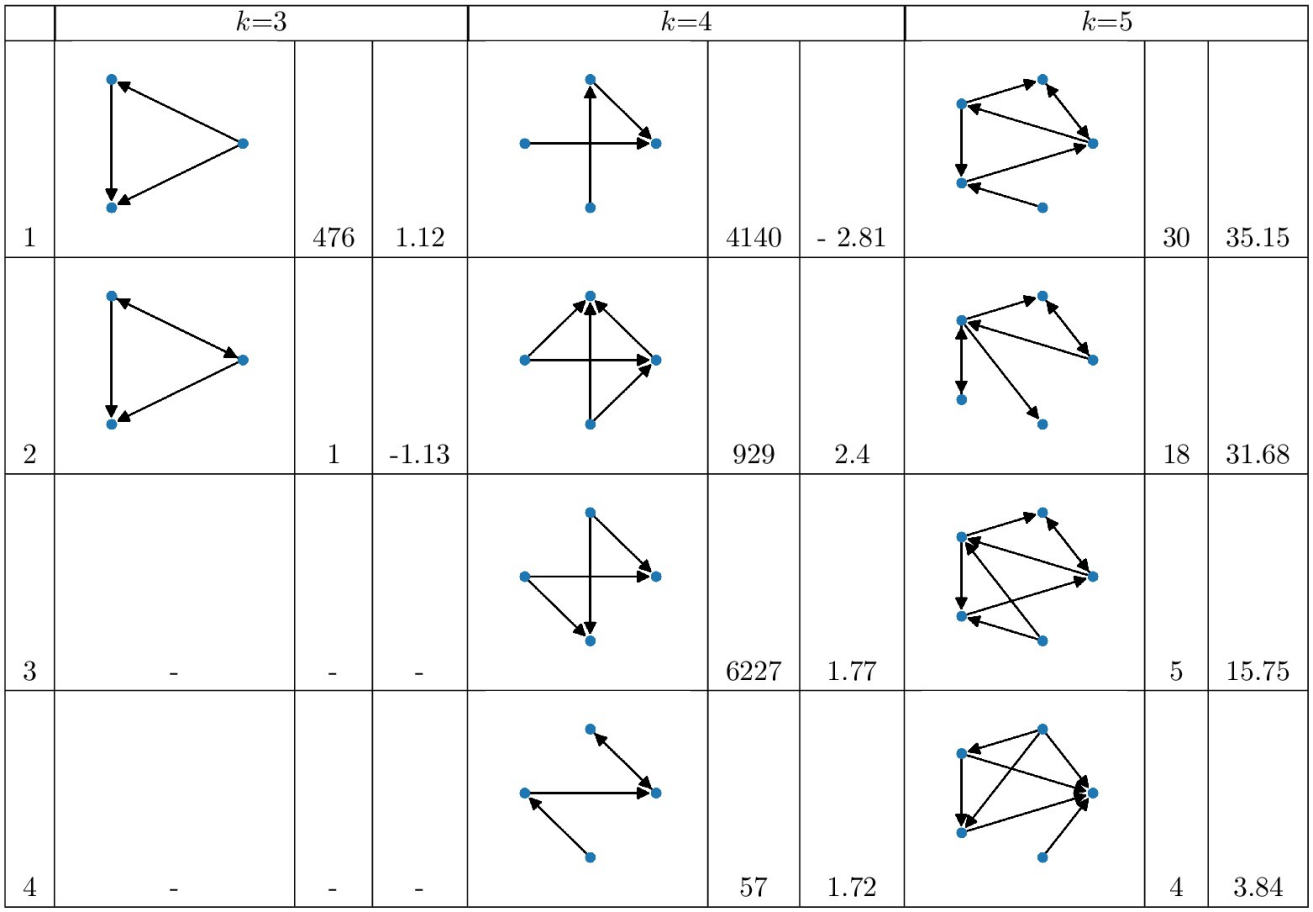
The four most significant network motifs on the *multi-sector* network for size *k* = 3, 4, 5. For each motif we report its numbers of occurrences and its *z-score*. For *k* = 3, just the first subgraph satisfies the criteria to be considered as significant, however we also reported the second most significant (under-represented) even if its frequency is 1.

In Fig 9 we reported the four most significant network motifs for size 3, 4 and 5 for the multi-sector network. In the *k* = 3 case, we identified only one over-represented network motif. The motif corresponds to the classical feed-forward loop, a scheme characterizing different biological and social networks. In fact, in this network we have a principal country investor which differentiates its deals into two countries, but one of them also invests in the other target country. This specific structure determines a rank among the members of the motif: a principal investor, a middle-country and a target. This deal pattern is quite frequent and it is worth noting that a similar pattern, where the relationships between the principal investor and the middle-country are symmetric, occurs just one time and it is under-represented, i.e. it would be more frequent in a null model of a land trade network. In the *k* = 4 case, we basically observe two types of network motifs. The first and the fourth motifs are essentially a composition of chains of deals. In the first one, we observe a country targeted by two chains, one direct and the other with a middle-country but without a feed-forward loop. This pattern is under-represented even if it is quite frequent, i.e. in a random model where we maintain the investment capacity of the countries we would expect this pattern to be more frequent. The second category of over-represented motifs (second and third subgraphs) captures the coupled behavior of two countries which invest in the same two target countries—they form a square. This behavior is very frequent and sometimes one of the two target countries invests in the other one. Finally, we postpone the discussion about the most significant motifs in the *k* = 5 case to Section 3.2 where we will compare the high-order structures of the multi-sector land trade network with the one observed in land trade network of specific investment sectors.

#### 3.1.1 Qualitative analysis

**Fig 10:**
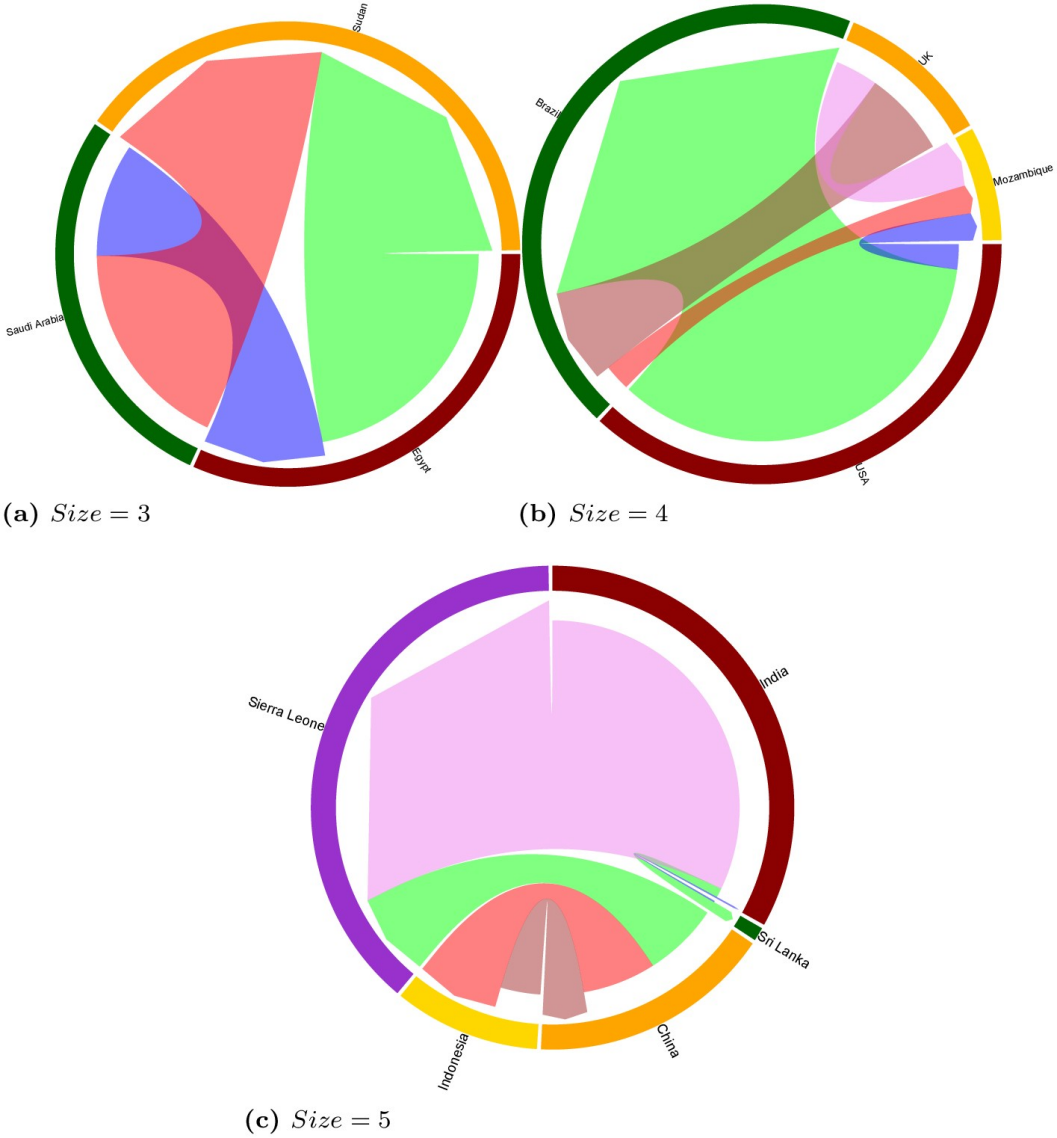
Chord diagrams visualizing examples motifs of size 3, 4 and 5 on the *multi-sector* network, with edge sizes proportional to edge weights (i.e., total size of the deals between two countries).

Even though we performed a complete analysis of statistically significant network motifs of size 3, 4 and 5 on the land trade network, here we focus the discussion on an example for each size. Fig 10 reports chord diagrams visualizing example motifs of size 3, 4 and 5, with directed edges going from the investor to the target country, and edge sizes proportional to edge weights (i.e., total size of the acquired land). Chord diagrams were produced by using the *circlize* R library. The aim of Fig 10 is to represent the interconnection between countries by insisting on the globalization of deals. More specifically, we selected notable examples of transnational land trade deals that allow us to discuss complex relations between different sub-regions of the world, like the ones between Africa and Middle-east in the motif of size 3, Global North and Global South in the motif of size 4, as well as relations among emerging economies (i.e., BRICS countries like India and China) with African countries and Southeastern Asia in the motif of size 5.

The example motif of size 3 (Fig 10a) shows a situation including one country (Saudi Arabia), investing in two neighbouring countries (Egypt and Sudan) to develop agricultural activities. One of those target countries also plays the role of investor, with agricultural activities being developed in the same target country, constituting a feed-forward loop. This situation exemplifies the relationship that countries with a high level of scarcity in arable lands display. Investments cascade towards countries with available lands or suitable environments for investment. This chord diagram also depicts the interest countries may have beyond acquiring resources. In this case, it shows the relationship between Saudi Arabia and other Muslim-majority countries situated in Africa. This motif shows a frequency of 476 and a *z-score* of 1.16 in the multi-sector network.

The example motif of size 4 (Fig 10b) represents a pattern involving two target countries (Brazil and Mozambique in the example), one of which is also investing in the other, and two countries that are investing in both target countries (UK and USA in the example). This motif has a frequency of 929 and a *z-score* of 2.4. The example network shows huge investments from USA to Brazil (14 deals which account for more than 532 000 ha). 82% of these account for investments in agriculture for bio-fuel and food crops (followed by mining and industry developments). UK is also investing in Brazil to relocate its agricultural production. Regarding Mozambique, the ultimate target country in this example, it receives balanced investments from three countries, which have targeted different sectors: forestry for carbon sequestration and timber plantations, agriculture for soybean, fruits, sugar cane, and mining for exploiting offshore gas fields.

As concerns the example motif of size 5 (Fig 10c), it represents five countries: one target country (Sierra Leone in the example), and four countries that are both investing and target countries (China, India, Indonesia and Sri Lanka in the example). This motif shows a frequency of 16 and a *z-score* of 8.9. In the situation shown in the example, Sierra Leone is receiving massive investments from India and China. India, through SIVA group, solely focuses on agriculture production on more than 120,000 ha, whereas China diversifies investments targeting mining, bio-fuel, bio-energy, and food crops, over 24,000 ha. Paradoxical situations can also be observed. For instance, China invests in Indonesia for agricultural production (24,000 ha), which sector contributes to high levels of deforestation in the country. On the other hand, Indonesia invests in China to develop timber plantations over 13,000 ha. The modest investment from Sri Lanka to India is accounted for the development of the textile industry.

### 3.2 Motifs on the *agriculture* and *mining* networks

To understand whether different trading behaviors and mechanisms affect the local structure of the networks related to different investment sectors, we compared the local structure of networks by adopting an approach based on the significance profile SP [68]. In the literature, there are two measures for the calculation of the significance profile of a network. The first method is based on a normalization of the *z*-score defined in Eq 2. In this case, the significance profile *SP* of a network is denoted by the vector < *NZ*(*D*_1_), …, *NZ*(*D*_*m*_) >, where the *k*-th element *NZ*(*D*_*k*_) is:

$$\begin{array}{c} NZ\left(D_{k}\right)=\frac{Z(D_{k})}{{\left(\sum_{i=1}^{m}Z{\left(D_{k}\right)}^{2}\right)}^{\frac{1}{2}}} \end{array}$$ (3)

where *m* is the total number of the network motifs we identified. Even if the mining and the agriculture networks are comparable in size, normalization makes the comparison more robust even in cases of different densities. The second method involves the use of the abundance Δ of a subgraph *D*_*K*_, defined as:

$$\begin{array}{c} \Delta \left(D_{k}\right)=\frac{f{\left(D_{k}\right)}^{G}-{\hat{f}}_{rand}\left(D_{k}\right)}{f{\left(D_{k}\right)}^{G}+{\hat{f}}_{rand}\left(D_{k}\right)+\delta } \end{array}$$ (4)

where *δ* is usually set to 4; since the normalized *z-score* for 4- and 5- network motifs shows a dependence on the size of the network [68]. Thus, from the above equation we define the subgraph ratio profile SRP [68], i.e. the vector < *N*Δ(*D*_1_), …, *N*Δ(*D*_*m*_) >, where the *k*-th element $N\Delta \left(D_{k}\right)=\Delta \left(D_{k}\right)/{\left(\sum_{i=1}^{m}\Delta {\left(D_{k}\right)}^{2}\right)}^{\frac{1}{2}}$.

In comparing the two networks we have applied both methods, but in the following analysis we focus on the results relating to the significance profile, since the main findings do not depend on the method used to evaluate the network profile and the size of the two networks is comparable.

**Fig 11:**
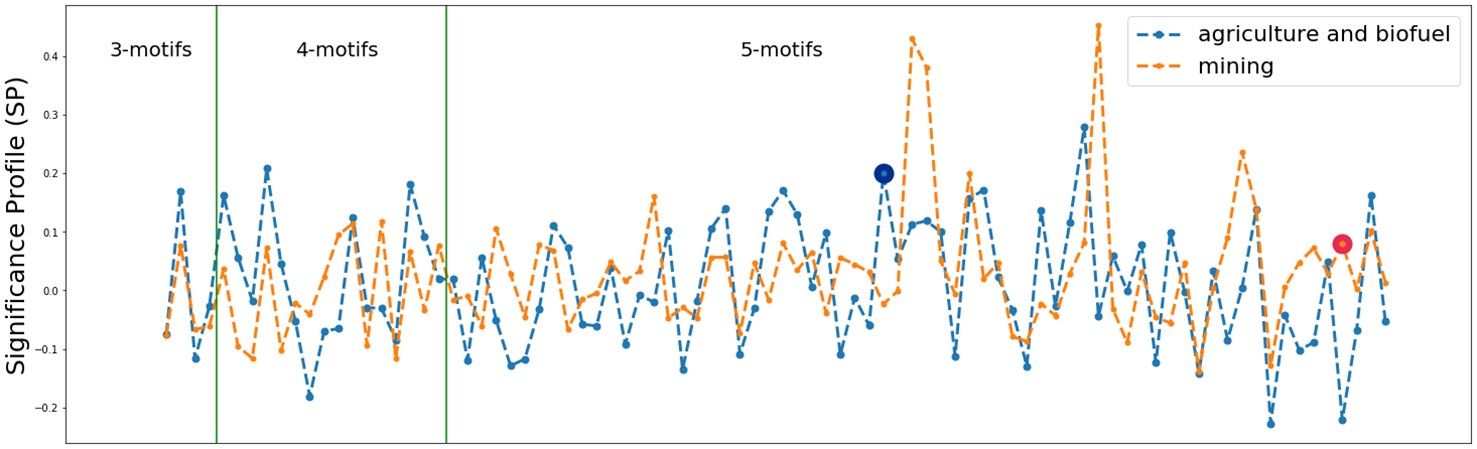
The significance profile SP based on 3-, 4-, and 5- network motifs with number of occurrences greater or equal to 4, common to both investment sectors. The orange line refers to the significance profile of the *mining* network, while the blue line refers to the *agriculture* network. The green lines group the network motifs according to their size. The blue and the red markers indicate the 5-network motifs whose normalized significance scores are very different between the investment sector networks.

**Fig 12:**
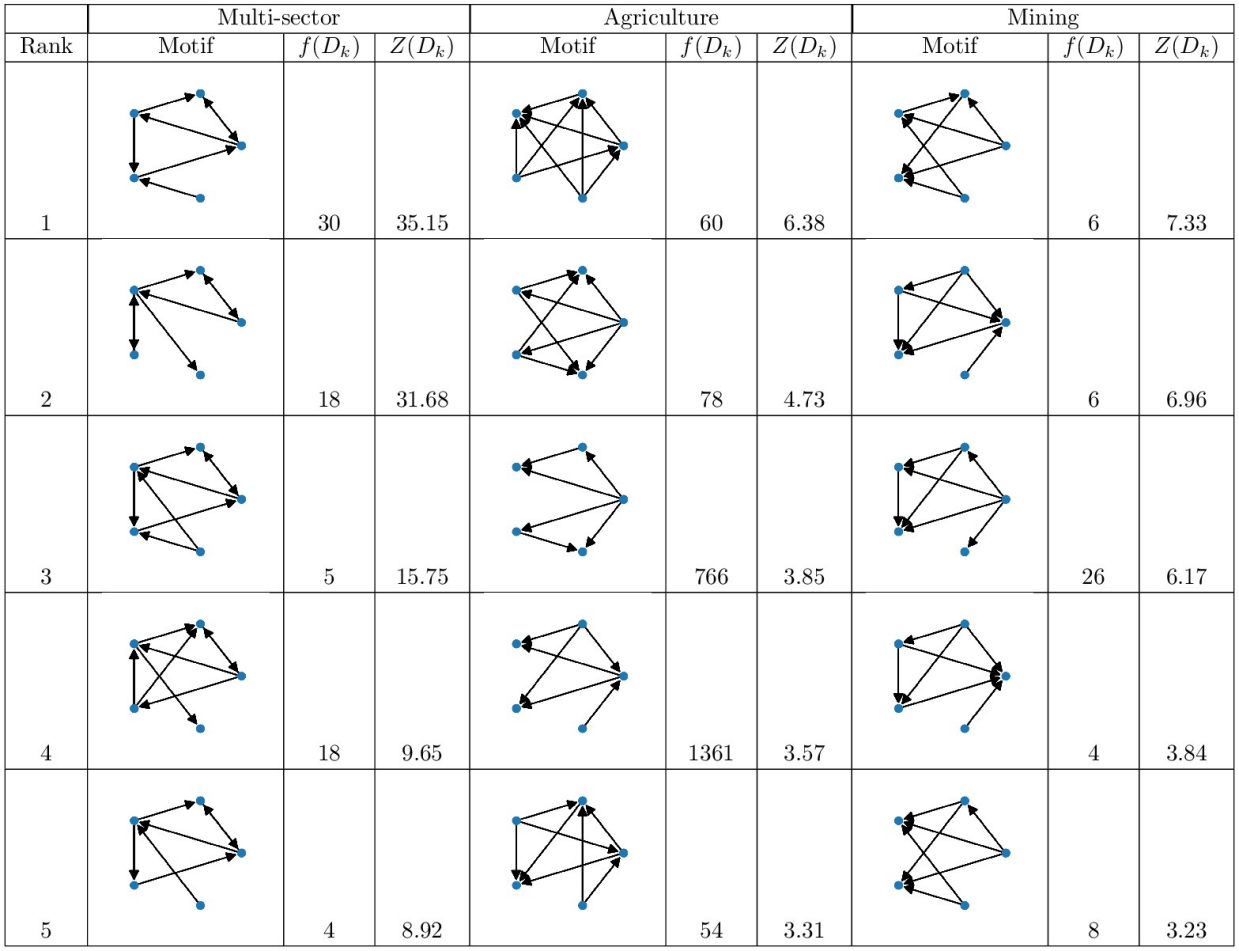
The five most significant network motifs for the *multi-sector*, *agriculture* and *mining* networks, respectively. For each motif we report its numbers of occurrences and its *z-score*.

In Fig 11 we have displayed the significance profile of the *agriculture* and *mining* networks based on the 3-, 4- and 5- directed network motifs. Specifically, for each investment sector we individuated the networks motifs whose number of occurrences is greater or equal to 4, then we identified the set of subgraphs common to both sectors. The significance profile of each network has been computed on the latter set, consisting of 89 subgraphs. From the figure, we observe that the significance profiles for 3-network motifs are quite similar, while the local structures of the two networks differ increasingly as the size of the subgraphs increases. To evaluate the difference between the two significance profiles we measure both the linear correlation by Pearson correlation coefficient *r* and the rank correlation by Kendall coefficient *τ*_*b*_ between the significance profiles. As for the linear correlation we obtained *r* = 0.27 (*p*-value < 0.01), while for the rank correlation we got *τ*_*b*_ = 0.22 (*p*-value < 0.005). Thus, the two networks of the agriculture and mining sectors are only weakly correlated. We can observe this weak correlation also in the data detailed in Fig 12. In the figure we report the five most significant network motifs—in terms of *z-score*—for the *multi-sector*, *agriculture* and *mining* networks along with their frequency and *z-score*. By inspecting and comparing the structure of the network motifs in the *multi-sector* network w.r.t. the other two networks, we observe that the five most significant motifs in the former are almost composed of triangle-shape triads which mainly form cycles and a few feed-forward loops. From the viewpoint of the trade network, the difference between a cycle and a feed-forward loop is quite important since they give the countries involved in the triad a different role. In the former, i.e. the cycle, each element is equally important since each country is the source and the destination of a deal at the same time, while in the latter, i.e. the feed-forward loop, there is a specific hierarchy where a node is an “initiator”—it is only a source of deals -, a node is a “target”—it is only a destination of deals—and an “intermediate” node which is both source and destination. Given this interpretation, the most significant network motifs in the *multi-sector* network are characterized by a less hierarchical structure, while in the *agriculture* and *mining* networks the most significant ones consist only of feed-forward loops, thus introducing a hierarchy and a specific role for the countries involved in the triads. The analysis of the structure of the motifs not only marks the difference between the multi-sector and the other two networks, but it also highlights the specificity of some motifs in the two investment sector networks. Specifically, all the triads constituting the motifs are feed forward loops (except for the fourth subgraph), through which we identify a principal “initiator” or at most two “initiators” who do not interact. In the case of the *mining* network, we noted *i*) a prevalence of motifs with a node marginal to the densest part of the subgraph (rank 2, 3 and 4), and *ii*) motifs made up by tetrads not decomposable into triads (rank 1 and 5).

Therefore, the lack of a strong correlation between the significance profiles of the *agriculture* and *mining* networks suggests that we are looking at two different types of network, each with specific motifs and anti-motifs and different high-order local structures that express different functions. Furthermore, the analysis of the composition of the most significant motifs in the three trade network has highlighted differences among them mostly related to motifs which clearly define roles in the motifs (agriculture and mining) and motifs that do not. These observations lead to the interesting possibility that the trading mechanisms and decisions which drive the evolution of these investment sectors are different and need to be the subject of a further investigation. Moreover, they underline the importance of analyzing the land trade network by differentiating the investment sectors.

#### 3.2.1 Qualitative analysis

**Fig 13:**
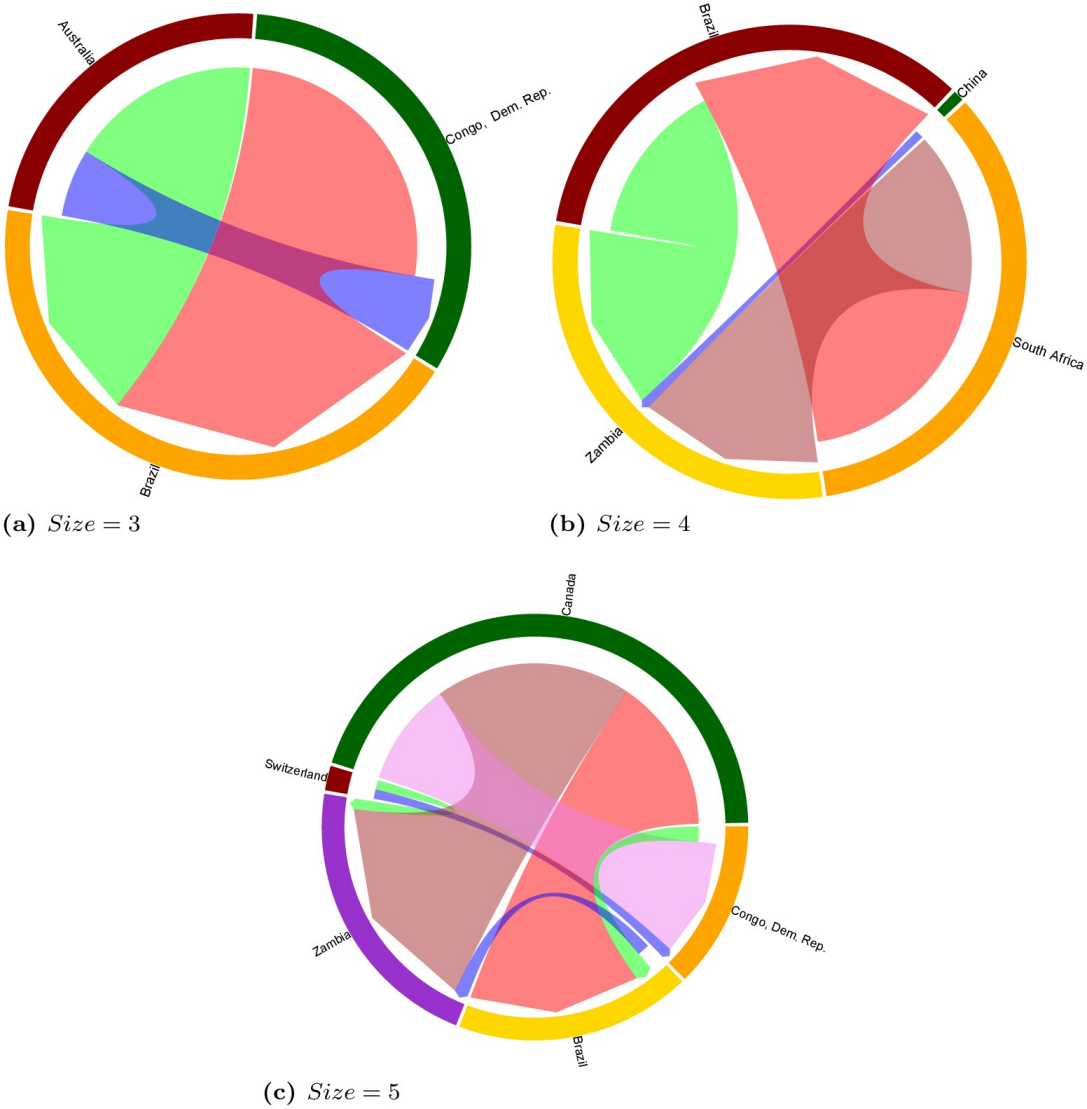
Chord diagrams visualizing examples motifs of size 3, 4 and 5 on the *mining* network, with edges sizes proportional to edge weights (i.e., total size of the deals between two countries).

**Fig 14:**
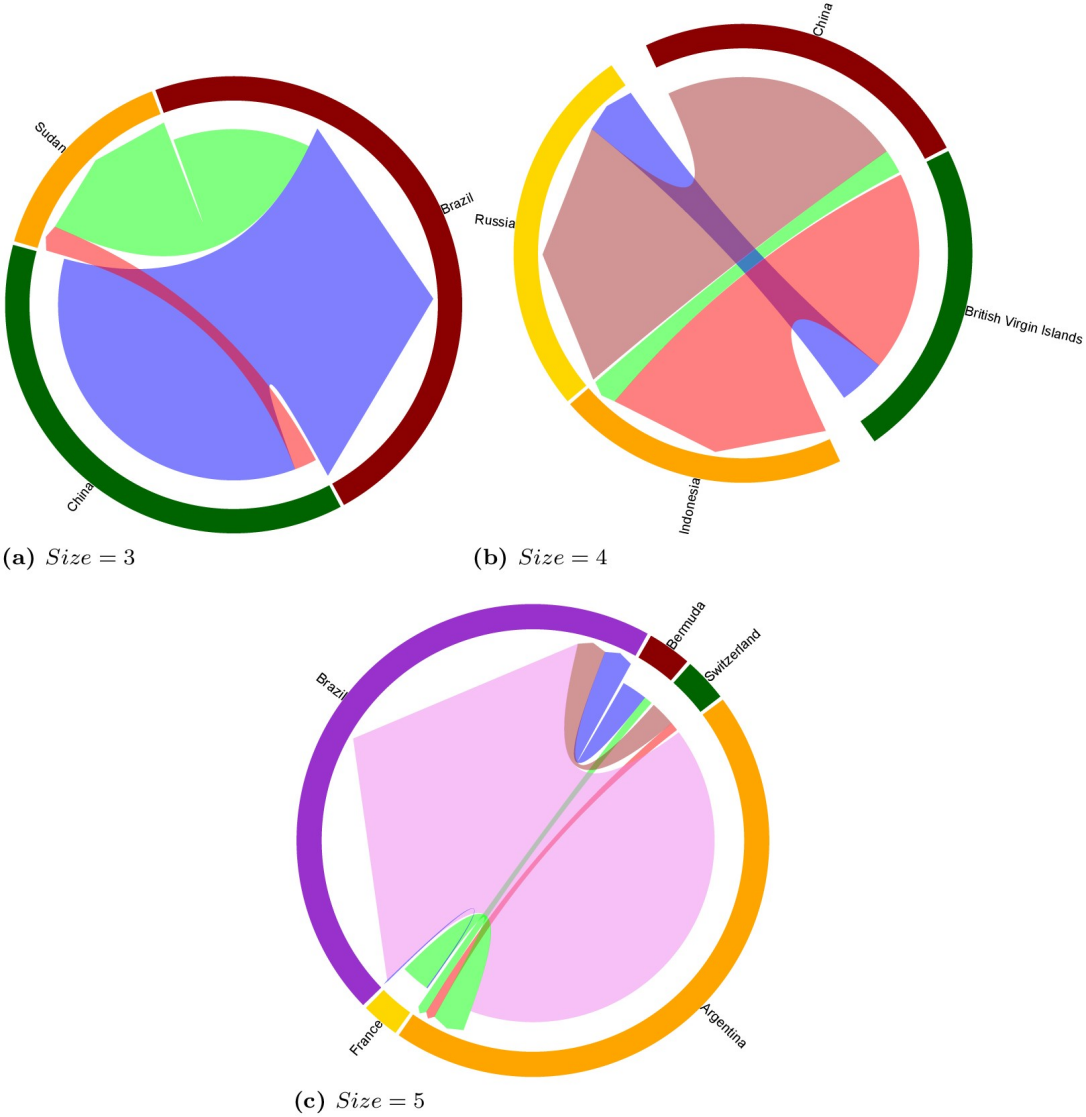
Chord diagrams visualizing examples motifs of size 3, 4 and 5 on the *agriculture* network, with edges sizes proportional to edge weights (i.e., total size of the deals between two countries).

As seen for the multi-sector network in Section 3.1.1, motifs can be a precious tool to discover complex relations between countries. For this reason, we resort again to a qualitative analysis of network motifs in order to provide a further insight in the agricultural and mining sectors, which are iconic in the current rush for resources. As done for the *multi-sector* land trade network (cf. Section 3.1.1), for each sector, we chose countries representative of the globalized nature of investments. For instance, Figs 13 and 14 display interactions at the scale of several continents.

*Mines network*. The example motif of size 3 (frequency of 13, *z-score* of 1.65) shows one investing country (Australia), one target country (Brazil) and one country both investor and target (Congo). In Congo, Australian company Tiger Resources Limited and national company La Générale des Carrière et des Mines, exploit a 5,500 ha mine for copper. In another part of the country, Australian MMG Limited is also involved in copper mining with a 1,610 ha acquisition. Australia, leading country in the mining sector, is also investing in Brazil for gold mining (over 20,000 ha). In partnership with South African company Anglo Gold Ashanti, the company Offices de Mines d’Or de Kilo Moto (OKIMO) from Congo is also present in Brazil for gold mining with a concession exceeding 30,000 ha.

The example motif of size 4 (frequency of 34, *z-score* of 1.23) is displaying two investing countries (South-Africa, China), one target country (Zambia) and one country both investor and target (Brazil). Zambia is experiencing investments from all three other countries for copper, cobalt and nickel, which are essentials for the batteries of electric vehicles. In relation to the motif size 3 above, Brazil receives investments for gold mining from South-Africa through Congo and the OKIMO company and through Anglo Gold Ashanti.

The example motif of size 5 (frequency of 6, *z-score* of 7.4) has two investing countries (Canada, Switzerland), one target country (Zambia) and two countries being both target and investor (Congo, Brazil). In this example, Canada plays an important role, investing in all three target countries. In Brazil, Canada’s company Largo Resources is involved in extracting Vanadium, toxic chemical compound for Human and environmental health, this metal is mainly used in pharmaceuticals and as an alloy. Another Canadian company, Cancana Resources Corp. is involved in Manganese mining, and four other companies from Canada are involved in gold mining in Brazil. A Congolese company is also associated with gold mining in Brazil over more than 30,000ha mine. On the Brazilian side, Vale S.A. is investing in Zambia with copper mining in Lubambe, and in association with companies from Zambia and Canada, Swiss company Glencore is involved in copper mining in Kitwe and Mufurila, Copperbelt Province in Zambia.

*Agriculture network*. The example motif of size 3 (frequency of 238, *z-score* of 2.42) is based on one target country (here, Sudan), one investing country (China) and one country, being both target and investing (Brazil). In Sudan, 60%–80% of the population is engaged in subsistence agriculture in a national context of economic and political crisis. In this context, Brazil is investing in irrigated agriculture over 12,000 ha along with the Arab Sudanese Blue Nile Agricultural Company. China is also investing in Sudan in genetically modified cotton, sunflower and peanut. In Brazil, China invests massively with more than 16,000 ha in food crops.

The example motif of size 4 (frequency of 204, *z-score* of 1.26) has two target countries (Russia and Indonesia) and two investing countries (China and the British Virgin Islands). Both investing countries invest in the two target countries. Listed in the Virgin Islands, Wellpoint Pacific Holdings Ltd is referenced in the panama papers and connected to other holding in Panama and Singapore. The database accounts for investments of up to 199,000 ha in Indonesian agriculture. Also originating from the Virgin Islands, Raysun International Corporation, listed in the Offshore leaks and tied to Honk Kong and Chinese interest is investing some 47,000 ha in Russia for food crops and livestock farming.

As regards the example motif of size 5 (frequency of 505, *z-score* of 2.17), three countries are investors (France, Bermuda and Switzerland), one country is a target for agricultural investments (Brazil) and one country is both target and investor (Argentina). In Brazil, Argentina, through two companies (El Tejar Ltd and Cresud S.A.) are responsible for 11 deals that are materialized by almost 500,000 ha used for food crops, livestock farming and biofuel. Bermuda, well know for harboring investment funds, hedge funds, trade and bank deposits is investing in both countries for 36,000 ha in the same value-chains, and through financial arrangements involving five different holdings, including one from Switzerland.

**Fig 15:**
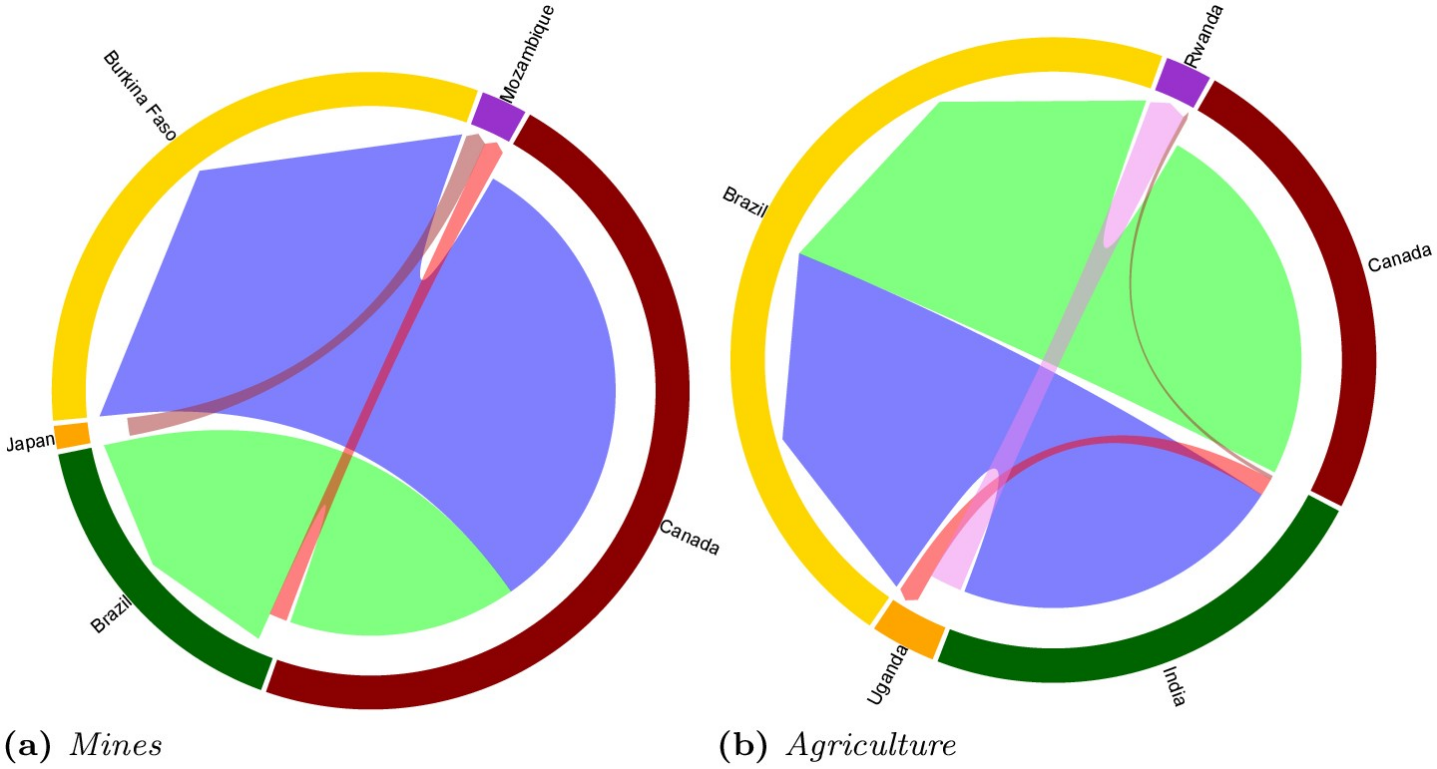
(a) Example of an occurrence of the 5-motif corresponding to the dark orange point in Fig 11. It is a significant subgraph in the *mining* network, but an anti-motif in the *agriculture* one. (b) Example of an occurrence of the 5-motif corresponding to the blue point in Fig 11. It represents a motif in the *agriculture* network, but an anti-motif in the *mining* one. In both chord diagrams the edges sizes is proportional to edge weights.

*Motifs vs. anti-motifs*. Finally, we complete our qualitative analysis on the motifs characterizing the two networks by leveraging the significance profiles computed in the previous section. Specifically, we computed the distance between each network subgraph common to *agriculture* and *mining* networks and among the subgraph with the highest distance we identified that one which is a motif in the agriculture sector and anti-motif in the mining one, and vice versa. The two relevant subgraphs are pointed out in Fig 11 by a blue (agriculture) and a dark orange(mining) markers. As regards the subgraph which is a motif in the *mining* network (*z-score* of 1.29) and an anti-motif in the *agriculture* network (*z-score* of −3.06), in Fig 15a we report an occurrence which involves Canada, Japan, Brazil, Mozambique and Burkina Faso. This network motif is characterized by a tree-like structure where we have a main investor (Canada), two main targets (Burkina Faso and Mozambique) and one country—Brazil—which is both a target and an investor, even if the amount of deals is heavily oriented towards in-going deals. In this example, Canada has developed 8 deals in Brazil through 6 different companies to mine vanadium, gold, manganese, and iron deposits. Conversely, in Fig 15b we display an occurrence of a motif which characterizes the *agriculture* network (*z-score* of 2.76) but not the *mining* one (*z-score* of −0.38). In this case we can decompose the subgraph into a feed-forward loop, where India is the initiator, Rwanda is the target and Uganda is the intermediate country; and a V-shape triad made up by India, Canada and Brazil, Brazil is the target and its in-going deals are equally split between India and Canada. Nine deals are registered for the Canadian alternative asset management company Brookfield, which diversifies investments in food crops, livestock farming and biofuel. Two deals from India, accounting for the same area targeted (over 100,000 ha), directed their investments towards a combination of biofuel and foodcrops.

## 4 Conclusion

### 4.1 Summary of findings

The results of the analyses carried out in this work show that (i) all continents are affected by the LSLA phenomenon, i.e., land investment dynamics are clearly global and (ii) both North-South dynamic (e.g. France invests in Vietnam) and South-South dynamic (e.g. Paraguay invests in Nigeria) hold in this context. The fact that an evident Global North-Global South dynamic characterizes the land trade market is a clear outcome of all our analysis stages. While the fact that land investment dynamics are global can be observed in the fact that the land trade networks are rather compact and dense (i.e., low average path length, average clustering coefficient), a first hint of the North-South dynamics already comes out from specific network properties (e.g., low reciprocity, low percentage of strongly connected node pairs, low assortativity) that show how the networks are characterized by a strong asymmetry of the deals.

In order to get an insight in these phenomena, we introduced the *LSLA-score*, a measure based on the total surface of acquired and sold land for each country which allows us to rank the countries based on their investor/target profile in the global land trade market. If from one side the analysis based on the *LSLA-score* (cf. Section 2) confirmed the Global North-Global South dynamic (also emphasizing the connection between investing countries showing low *LSLA-score* values and members of the G20 and Paris Club groups), from another one it allowed to discover the importance of emerging economies (i.e., the BRICS countries). While the majority of target countries (corresponding to high values of *LSLA-score*) is actually located in the Global South (due to characteristics such as appropriate climate conditions, profitable labor force, available arable land and water), sub-regions like Latin America and Sub-Saharan Africa also account for a significant total quantity of acquired land thanks to emerging economies like Brazil and South Africa (showing medium values of *LSLA-score*, due to the fact that, even if they tend to have an *investor* profile, they are also involved as target countries in several deals). The analysis of *LSLA-score* on the *mining* land trade network also allowed us to find how mining land deals are mainly located in Central America, South America, Africa and South Asia, where the Global North and BRICS are relocating extraction of minerals and related pollution. Conversely, we noted how in the *agriculture* network the importance of the BRICS was strongly reduced, while the leading investors included countries from North America, Western Europe and Gulf states (i.e. Saudi Arabia and the United Arab Emirates).

Our analysis also demonstrated how land investments dynamics are strongly correlated with country development indicators. We showed how the top investing countries are characterized by higher development (i.e., higher values of CPI, HDI and GNI) and generally correspond to G20 countries. Conversely, the main target countries are the ones characterized by low levels of governance and high levels of corruption and food insecurity. Moreover, we found an interesting correlation between *biocapacity* and *betweenness*, possibly indicating that the countries characterized by high values of biocapacity may play the role of *flow hubs* in the land trade network.

With the aim to deepen our knowledge about complex land trade schemes among countries, we also performed an extensive analysis based on high-order structures, i.e., network motifs of size 3, 4 and 5 on the three land trade networks (cf. Section 3). Quantitative analysis showed how, consistently with previous analyses, the most significant network motifs in the *multi-sector* network are characterized by a less hierarchical structure (e.g., *cycles* where each country can be investor and target at the same time), while in the *agriculture* and *mining* networks the most significant motifs consist mainly of feed-forward loops, thus introducing a hierarchy and a specific role for the countries involved in the triads (i.e., an *initiator* which is only a source of deals, a *target* which is only a destination of deals, and an *intermediate* country which is both source and destination). Quantitative comparison between the significance profiles of the motifs on the three networks also confirmed how the trading mechanisms and decisions which drive the evolution of the *agriculture* and *mining* investment sectors are different both among them and with respect to the *multi-sector* case, thus underlining the importance of analyzing the land trade network by differentiating the investment sectors. Finally, qualitative analysis of network motifs allowed us to focus on notable examples of transnational land trade deals, carrying out detailed discussions over relations among different sub-regions of the world, such as Africa and Middle-east, Global North and Global South, relations connecting emerging economies to African countries and Southeastern Asia, as well as interactions at the scale of several continents.

### 4.2 Concluding remarks and future work

In this work we focused on the global problem of large scale land acquisitions, by modeling three land trade networks out of the data contained in the Land Matrix Initiative database: a *multi-sector* network, a network centered on the *mining* sector, and a network centered on the *agriculture* one. These networks were analyzed by means of state of the art network analysis techniques, with the aim to characterize the behavior of different countries on the global land trade market, and to discover recurrent patterns in the land trade dynamics. To this purpose, we defined a score, namely *LSLA-score*, which proved to be effective in ranking the countries based on their investing/target role in the land trade network. Leveraging on this network representation, we analyzed the land trade scenario from a quantitative and qualitative point of view, also performing an analysis based on the correlation between centrality measures and different country development indicators. We also resorted to network motifs (i.e., recurring, statistically significant subgraphs), in order to provide an insight into higher order correlations between countries, thus providing original knowledge about recurring patterns in transnational land trade deals. Our analyses showed how the land trade market is massively characterized by the Global North-Global South divide, that the market is global and involves deals among all sub-regions of the world, and that the rise of emerging economies like the BRICS is a phenomenon that is likely to influence the land trade market more and more in the years to come. Moreover, the analyses on specific sectors such as mining and agriculture highlighted how, for several countries, the role in the trade network and the relation patterns with other countries can drastically change when taking into account different investment sectors, and that hierarchies between investor, intermediate and target countries can be stronger in specific investment sectors (with respect to the *multi-sector* case).

While this work showed how large scale land acquisitions can be effectively studied by leveraging on complex network analysis and mining techniques, research in this domain can still be advanced by addressing several future challenges. Since the results produced by the use of network motifs on the land trade network were highly promising, a future investigation may regard a deeper qualitative analysis of such results. For instance, while in this work we focused on deals in operation, we can suppose that notable statuses of the implementations (e.g., abandoned ones) may show correlation with specific network motifs. Another challenge regards the introduction of the temporal dimension in the land trade analysis. Temporal information about the deals contained in the Land Matrix database can be exploited to discover temporal sequences associated to specific categories of deals (e.g., successful/unsuccessful ones). The analysis of the network motifs in the three different networks taken into account in this work (i.e., *multi-sector*, *agriculture* and *mining*) also opens to the possibility of obtaining a hierarchy of the countries based on their position in the motifs, i.e., by inferring from the network motifs their role in different land trade networks (e.g., investor, intermediate, target). Temporal information may also be used to further characterize countries based on the evolution of their behavior on the land trade market in different time periods. For instance, a time series of *LSLA-score* calculated for each country at different time stamps would allow to exploit classic time series clustering algorithms in order to discover countries with similar trading profiles.

Further research steps may regard the modeling of the land trade network. The rich information provided by the Land Matrix would allow to go beyond the country-based network studied in this work, by exploiting, at modeling time, complex relations among a wider set of actors, such as investors, operating companies and top companies. Finally, the characterization of LSLAs could be further improved by resorting to alternative sources of information, which may include not only online media (e.g., social network data, online press), but also data coming from investigative journalism (e.g., data from paradise/panama papers), that may lead to the discover complex relations between the actors that go beyond the land trade market.
